# GAGE: generally applicable gene set enrichment for pathway analysis

**DOI:** 10.1186/1471-2105-10-161

**Published:** 2009-05-27

**Authors:** Weijun Luo, Michael S Friedman, Kerby Shedden, Kurt D Hankenson, Peter J Woolf

**Affiliations:** 1 Department of Biomedical Engineering, University of Michigan, Ann Arbor, MI 48109, USA; 2 Bioinformatics Shared Resource, Cold Spring Harbor Laboratory, Cold Spring Harbor, NY 11724, USA; 3 Thermogenesis Corporation, Rancho Cordova CA, 95742, USA; 4 Department of Statistics, University of Michigan, Ann Arbor, MI 48109, USA; 5 Department of Animal Biology, University of Pennsylvania, Philadelphia, PA 19104, USA; 6 Bioinformatics Program, University of Michigan, Ann Arbor, MI 48109, USA; 7 Department of Chemical Engineering, University of Michigan, Ann Arbor, MI 48109, USA

## Abstract

**Background:**

Gene set analysis (GSA) is a widely used strategy for gene expression data analysis based on pathway knowledge. GSA focuses on sets of related genes and has established major advantages over individual gene analyses, including greater robustness, sensitivity and biological relevance. However, previous GSA methods have limited usage as they cannot handle datasets of different sample sizes or experimental designs.

**Results:**

To address these limitations, we present a new GSA method called Generally Applicable Gene-set Enrichment (GAGE). We successfully apply GAGE to multiple microarray datasets with different sample sizes, experimental designs and profiling techniques. GAGE shows significantly better results when compared to two other commonly used GSA methods of GSEA and PAGE. We demonstrate this improvement in the following three aspects: (1) consistency across repeated studies/experiments; (2) sensitivity and specificity; (3) biological relevance of the regulatory mechanisms inferred.

GAGE reveals novel and relevant regulatory mechanisms from both published and previously unpublished microarray studies. From two published lung cancer data sets, GAGE derived a more cohesive and predictive mechanistic scheme underlying lung cancer progress and metastasis. For a previously unpublished BMP6 study, GAGE predicted novel regulatory mechanisms for BMP6 induced osteoblast differentiation, including the canonical BMP-TGF beta signaling, JAK-STAT signaling, Wnt signaling, and estrogen signaling pathways–all of which are supported by the experimental literature.

**Conclusion:**

GAGE is generally applicable to gene expression datasets with different sample sizes and experimental designs. GAGE consistently outperformed two most frequently used GSA methods and inferred statistically and biologically more relevant regulatory pathways. The GAGE method is implemented in R in the "gage" package, available under the GNU GPL from .

## Background

A central goal of biomedical research is to define mechanistic causes for cellular behavior and disease. High throughput technologies such as gene expression profiling provide a rich starting point to identify mechanistic causes, e.g. de novo network inference [1]. Ideally we would like to contextualize gene expression patterns with the known biochemical processes and regulatory signaling pathways. This approach provides us with a more systems level and informative view (compared to individual gene based interpretation) of the biological states that have been perturbed, which in turn allows us to identify points where we could intervene to change cellular behavior.

Gene set analysis (GSA) is a widely used strategy for gene expression data analysis based on pathway knowledge [2-12]. Unlike previous strategies which focus on individual or a limited number of genes, GSA focuses on sets of related genes and has demonstrated three major advantages. First, GSA methods are better able to detect biologically relevant signals and give more coherent results across different studies [3,5]. Second, GSA uses all of the available gene expression data (cutoff-free) instead of prefiltering the data for a short list of strongly differentially expressed genes (cutoff-based). (Note that cutoff-based tools such as WebGestalt [13] and FatiScan [14] that apply Fisher's test and Hypergeometric test are sometimes denoted as gene set analysis tools.) Indeed, small coordinated gene expression changes in a pathway can have a major biological effect even if these changes are not statistically significant for any individual gene [3]. Third, GSA incorporates prior knowledge of biological pathways and other experimental results in the form of gene sets [3,4]. These gene sets are constantly updated in the literature and represent a significant repository of useful biological knowledge. Although, knowledge dependency can be also considered a limitation of GSA strategy: our findings are restricted by current knowledge.

There are two categories of GSA based on the statistical tests used: sample randomization and gene randomization [8,15]. Sample randomization methods test significance of gene sets based on permutation of sample labels, with GSEA [3,4], SAFE [10] and SAM-GS [9] as representatives. In contrast, gene randomization methods test the significance of gene sets based on permutations of gene labels or a parametric distribution over genes, with PAGE [5], T-Profiler [7] and Random-set [6] as representatives. Sample randomization maintains the correlation structure among genes but only applies to large expression datasets with multiple samples per experimental condition. For a two-state comparison, a minimum of 8 chips for each state is required for 1000 balanced (presence of the two sample states) permutation or 6 chips for 1000 unbalanced permutation. Gene randomization has no limitation on sample size, but may break the correlation structure among genes [11]–an issue that may or may not be a problem (detailed in discussion) [5,6]. Sample randomization and gene randomization test different but related null hypotheses, Tian *et al*. [8] and Nam *et al*. [15] proposed combinatory procedures to achieve more robust results.

All these methods established GSA as a powerful strategy for gene expression data analysis. In spite of its advantages, GSA as a whole strategy still suffers from three major limitations.

First, currently available GSA methods do not handle small datasets effectively, yet most gene expression datasets fall into this category. As mentioned above, the sample randomization strategy used by methods such as GSEA is not appropriate for studies with under 8 gene chips per state, thus gene randomization remains to be the only feasible option [3,15]. Gene randomization methods such as PAGE have been applied to small dataset [5], but these methods tend to make large number of (false) positive calls with extremely small p-values [16,17] (also see the results). T-profiler targets datasets with one sample pair [7], however, it can't combine results from multiple paired experiments nor can it be applied to studies with non-paired studies [7].

Second, no GSA method currently available handles datasets with different sample sizes and experiment designs consistently. For datasets with few or no replicates, t-test statistics, signal to noise ratios, or their corresponding p-values are not robust estimates of differential expression for genes or simply not applicable. Therefore, fold change (log based) is frequently used as more versatile per gene statistics [3,5-7,18]. The use of fold change gives rise to two issues that have been largely neglected so far. First, the average fold change does not account for different experimental designs, i.e. pair-matched samples or non-paired samples. The per gene statistics such as t-test statistics may vary significantly depending on if the samples are paired or not, yet there is no difference in fold change. Second, average fold change does not contain any information for the sample size. Sample size largely determines the confidence or significance level of our inference, yet is dropped when using fold change. Fold change makes sense in one-on-one paired comparison, as in T-profiler [7]. However for datasets with replicate samples, the test power or the significance of relevant gene sets would be underestimated.

Third, most GSA methods only consider transcriptional regulation in one direction (e.g. all up or all down) in a gene set. This directional bias makes sense for experimentally derived gene sets, but not for gene sets based on canonical signaling pathways, which frequently show reciprocal gene regulation in both directions upon perturbation [19,20]. Thus it is advisable to consider both cases for an inclusive analysis for regulatory mechanisms.

**Figure 1:**
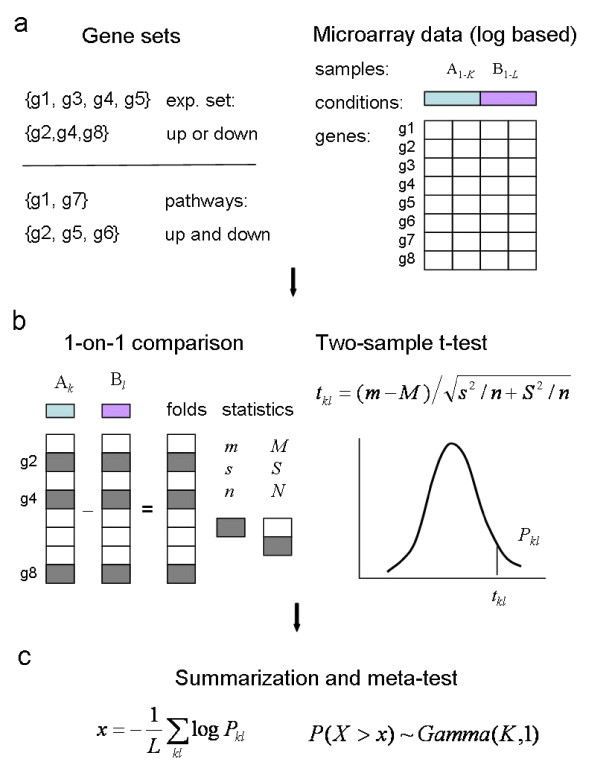
**A schematic overview of the GAGE algorithm. GAGE has three major steps**. (a) Step 1: input preparation. Separate gene sets into two categories: experimental sets and canonical pathways, for differential treatment in significant test. (b) Step 2: gene set differential expression tests based on one-on-one comparison between samples from the two experimental conditions. For each experiment-control pair, calculate differential expression in log based fold change for all genes. Test whether specific gene sets are significantly differentially expressed relative to the background whole set using two-sample t-test. (c) Step 3: summarization. For each gene set, derive a global p-value based on a meta-test on the negative log sum of p-values from all one-on-one comparisons. More details of GAGE are given in the Methods. Variables *m*, *s *and *n *are the mean fold change, standard deviation and number of genes in a gene set, *M*, *S *and *N *are those for the whole set. A similar schematic overview of the PAGE algorithm is shown in Additional file 1: Supplementary Figure 1.

To address these issues, we have developed a novel method called Generally Applicable Gene-set Enrichment (GAGE) (Figure 1). GAGE applies to datasets with any number of samples and is based on a parametric gene randomization procedure. Similar to Parametric Analysis of Gene Set Enrichment (PAGE) [5] (Additional file 1: Supplementary Figure 1) and T-profiler [7], GAGE uses log-based fold changes as per gene statistics. However, GAGE differs from PAGE and T-profiler in three significant ways. First, GAGE assumes a gene set comes from a different distribution than the background and uses two-sample t-test to account for the gene set specific variance as well as the background variance. In contrast, PAGE assumes gene sets comes from the same distribution as the background and uses one-sample z-test that only considers the background variance [5]. T-profiler also employs two-sample t-test, but it is essentially a one-sample z-test since the sample size of a gene set is not comparable to its complementary set [7] (Additional file 1: Supplementary Note 1 and Methods). Second, GAGE adjusts for different microarray experimental designs (paired or non-paired) and sample sizes by decomposing group-on-group comparisons into one-on-one comparisons between samples from different groups. GAGE derives a global p-value using a meta-test on the p-values from these comparisons for each gene set. Third, GAGE separates experimentally perturbed gene sets (from literature) and canonical pathways (from pathway databases). Experimental sets are taken as genes coregulated towards a single direction, whereas canonical pathways allowed changes in both directions. This gene set separation strategy give GAGE more test power in detecting relevant biological signals.

In this work, we show that GAGE is generally applicable to datasets with different sample sizes and experimental designs. We first apply GAGE to two lung cancer datasets [21,22] and one type 2 diabetes dataset [4], which has been analyzed by GSEA [3,4] and PAGE [5] as example cases. These are representatives for large datasets with tens of samples per condition frequently seen in large clinical or experimental studies. We then analyze a smaller, previously unpublished dataset describing mesenchymal stem cell response to BMP6 treatment. This is a typical small dataset with as few as two samples per condition like in most experimental studies. BMP6 treated samples and controls are one-on-one matched, which is a frequently used experiment design particularly for all the two-channel microarray studies. In each case, we compare GAGE to GSEA and PAGE. To compare the performance of GAGE vs GSEA and PAGE in a more controllable setting, we conducted simulation study using the type 2 diabetes dataset and synthetic gene sets. Finally, we also detail the major strategies employed by GAGE.

## Results

### Application to large datasets with the GSEA and PAGE as control methods

As a test case, we applied GAGE, PAGE and GSEA to two lung cancer datasets [21,22] which were originally analyzed and compared by GSEA [3]. These two datasets were generated by two independent studies done in Boston [22] and Michigan [21], containing gene expression profiles of lung adenocarcinomas samples from patients. Patients were classified as having "good" or "poor" clinical outcomes. For each dataset, we defined the control set as patient profiles with good clinic outcomes, and selected the most differentially regulated gene sets associated with poor outcomes. Note that we used the updated curated gene set collection c2 from MSigDB [3,23] for both methods. For a fair comparison, experimental sets and the canonical pathways were separated for all three methods.

We compared the top 10 most significant gene sets inferred by the three methods (Table 1 and 2, Additional file 1: Supplementary Table 1–3) and identified evident differences in four aspects. First, the top experimental gene sets selected by GAGE and PAGE overlapped significantly, but the canonical pathways identified by GAGE, PAGE, and GSEA did not (Additional file 1: Supplementary Table 3). The lack of overlap for the canonical pathways is expected because GAGE allows perturbations in both directions in canonical pathways. Second, GAGE derived modest p-values and numbers of significant gene sets compared to GSEA and PAGE (Table 2). While others have suggested that GSEA suffers from low sensitivity [5,8,9], our results suggest that PAGE is overly sensitive (low specificity). Third, the top 10 gene sets inferred by GAGE are more consistent between the two studies: 4 experimental sets and 5 canonical pathways are the same for GAGE results, 4 and 4 for PAGE and 1 and 0 for GSEA respectively (Table 2). Fourth, the top 10 gene sets inferred by GAGE better describe poor outcomes of lung cancer mechanistically (Table 2). Canonical pathways inferred by GAGE are by far the most indicative of tumor occurrence and metastasis. Experimental sets inferred by GAGE and by PAGE are similarly indicative of tumor occurrence and prognostic of metastasis or poor clinical outcomes, and both are better than those inferred by GSEA.

**Table 1:** GAGE applied to the two lung cancer datasets of large sample sizes

Boston study				Michigan study			
Experimental Sets	p-val	q-val	Notes	Experimental Sets	p-val	q-val	Notes

**Tarte_Plasma_Blastic**	1.8E-64	1.1E-61	c	**Tarte_Plasma_Blastic**	5.6E-42	4.1E-39	c
**Uvb_Nhek3_All**	1.2E-59	3.6E-57	t	**Cancer_Undifferentiat**	1.0E-22	3.8E-20	bt
Peng_Glutamine_Dn	3.7E-59	7.6E-57	c	**Brca_Er_Neg**	8.3E-19	2.0E-16	bt
Lei_Myb_Regulated_G	5.8E-55	8.8E-53	bt, c	Serum_Fibroblast_Cell	3.2E-18	5.9E-16	bt, c
Peng_Leucine_Dn	4.0E-42	4.8E-40	c	**Uvb_Nhek3_All**	5.3E-17	7.7E-15	t
**Cancer_Undifferentiat**	3.0E-41	3.0E-39	bt	Caries_Pulp_Up	4.7E-16	4.6E-13	
**Brca_Er_Neg**	2.0E-40	1.7E-38	bt	Zhan_Mm_Cd138_Pr_	8.3E-15	1.0E-12	bt
Peng_Rapamycin_Dn	3.5E-38	2.7E-36	c	Li_Fetal_Vs_Wt_Kidne	3.7E-14	3.8E-12	t
Rcc_Nl_Up	5.2E-36	3.5E-34	t	Dox_Resist_Gastric_Up	1.2E-13	1.1E-11	bt
Cancer_Neoplastic_Me	4.2E-35	2.6E-33	t	Idx_Tsa_Up_Cluster3	2.4E-13	1.9E-11	c

Canonical Pathways	p-val	q-val	Notes	Canonical Pathways	p-val	q-val	Notes

**Gpcrs_Class_A_Rhod**	9.2E-23	3.1E-20	bt	**Gpcrs_Class_A_Rhod**	3.1E-10	1.0E-07	bt
**Gpcrdb_Class_A_Rho**	4.7E-21	7.8E-19	bt	**Gpcrdb_Class_A_Rho**	1.1E-09	1.9E-07	bt
**Blood_Clotting_Casca**	5.1E-15	4.7E-13	bt	Androgen_Genes	5.2E-08	5.8E-06	bt
**Intrinsicpathway**	6.3E-15	5.3E-13	bt	Cytokinepathway	1.9E-07	1.6E-05	bt
Fibrinolysispathway	1.1E-12	9.1E-11	bt	Prostaglandin_And_Le	2.9E-05	2.4E-03	bt
**Peptide_Gpcrs**	1.9E-12	1.6E-10	bt	Proliferation_Genes	5.1E-05	4.3E-03	c
Tyrosine_Metabolism	8.7E-09	7.3E-07	bt	**Peptide_Gpcrs**	5.8E-05	4.8E-03	bt
Extrinsicpathway	5.5E-07	4.6E-05	bt	**Intrinsicpathway**	9.1E-05	7.6E-03	bt
Gpcrdb_Other	5.2E-06	4.4E-04	bt	Androgen_And_Estrog	4.2E-04	3.4E-02	bt
Small_Ligand_Gpcrs	6.7E-06	5.6E-04	bt	**Blood_Clotting_Casca**	7.5E-04	5.9E-02	bt

Top 10 most significantly enriched experimental sets and canonical pathways in poor clinical outcomes vs good outcomes were inferred by GAGE from two published lung adenocarcinoma data sets used in the GSEA paper [3]. Both positively and negatively regulated gene sets were collected and ranking by the p-value, and by absolute value of average t-statistics (data not shown) for ties. FDR q-values were estimated to correct the p-values for the multiple testing issue. Consistencies between the two data sets are shown in bold font. Notes show the connections of the gene sets to cancer related topics: t for tumor related; bt for tumor metastasis and bad outcome; c for cell growth and proliferation related; and blank represents no evident connection. These annotations came from the original studies for experimental sets, or made based on more than three independent literature works for the canonical pathway.

**Table 2:** Comparison between GAGE, PAGE and GSEA results from the two lung cancer datasets

Gene Sets & Methods		Overlap	Top 10 p-values	Metastasis	Tumor	Sign. Sets
Experimental Sets	GAGE	4	4.2E-35, 2.4E-13	3, 5	6, 7	242 (283), 120 (124)
	
	PAGE	4	1.0E-170, 2.0E-85	6, 4	8, 6	698 (757), 585 (655)
	
	GSEA	1	5.7E-3, 6.4E-3	1, 2	6, 4	3 (0), 4 (0)

Canonical Pathways	GAGE	5	6.7E-6, 7.5E-4	10, 9	10, 9	20 (16), 10 (8)
	
	PAGE	4	4.2E-26, 3.7E-27	2, 3	4, 3	170 (202), 153 (186)
	
	GSEA	0	1.1E-2, 1.4E-2	1, 1	5, 5	2 (0), 4 (0)

The top 10 most significantly enriched experimental sets and canonical pathways in poor clinical outcomes vs good outcomes were inferred by GAGE, PAGE, and GSEA from two published lung adenocarcinoma data sets used in the GSEA paper [3]. Data columns are overlap between top 10 gene sets for the two studies, top 10 p-values, number of top 10 gene sets related to metastasis (bt) and tumor (t and bt), and numbers of significant gene sets with p-value ≤ 0.001 (or FDR q-value ≤ 0. 01).

Several major mechanistic themes predictive of poor clinical outcomes emerged from the list of top gene sets inferred by GAGE. These themes included G-protein coupled receptors (GPCRS) associated signals (sets 1, 2, 6, 9, 10 of Boston and sets 1, 2, 7 of Michigan in Table 1), thrombosis or blood coagulation activation (sets 3, 4, 5, 8 of Boston and set 8, 10 of Michigan in Table 1), and hormone and cytokine (sets ranking >10 of Boston not shown, and set 3, 4, 9 of Michigan in Table 1). Indeed, G-protein-coupled receptors, the largest family of cell-surface molecules involved in signal transmission, have emerged as crucial players in the growth and metastasis of multiple human cancers [24,25]. Thrombosis or blood coagulation activation has been implicated in the disease and is an predictor for poor survival rates for lung cancer patients [26,27]. Androgen level and cytokine profiles influence clinic outcomes of non-small cell lung cancer [28,29]. All these factors are likely the major causal or contributing mechanisms for non-small cell lung cancer progress and metastasis.

We also applied GAGE, PAGE and GSEA to another large dataset describing type 2 diabetes progression that was analyzed by GSEA [4] and PAGE [5] previously (Additional file 1: Supplementary Table 6–7 and Supplementary Note 2). This comparison performed similarly to the cancer study mentioned above. In particular, GAGE pinpointed multiple experimental sets and canonical pathways which are directly involved in type 2 diabetes or closely related metabolism processes.

### Application to small datasets with PAGE and GSEA-g (GSEA with gene permutation option) as control methods

We applied GAGE and PAGE to a microarray dataset generated by our group to select the most differentially expressed gene sets in human mesenchymal stem cells (MSC) upon BMP6 treatment (Table 3 and 4, Additional file 1: Supplementary Table 8). The dataset contains a total of 4 gene chip measurements from duplicate experiments each with paired measurements of human MSC with or without 8 hours BMP6 treatment. Note that GSEA by default is not applicable to this dataset because the sample size is too small for permutation based inference. However, GSEA with gene labels permutation option (GSEA-g) works. Since GSEA-g does not implement the sample randomization strategy recommended by the authors [3], we focus on comparing GAGE to PAGE here (Table 4, Additional file 1: Supplementary Table 9–10). GAGE conducts one-on-one comparisons, hence was applied to each of the two BMP6 experiments individually (Table 3). For an exact comparison, PAGE was slightly modified to enable one-on-one comparisons (Additional file 1: Supplementary Table 8). The GSEA software took multiple samples per condition hence not applicable to the experiments individually (Additional file 1: Supplementary Table 9).

**Table 3:** GAGE applied to the BMP6-MSC dataset of small sample size

Experimental Sets	t-statistic	p-value	q-value	P.exp1	P.exp2
Ifna_Hcmv_6hrs_Up	-3.80	2.9E-07	2.9E-04	3.7E-04	1.6E-04
Der_Ifnb_Up	-3.47	1.6E-06	8.1E-04	3.3E-03	1.1E-04
Baf57_Bt549_Dn	-3.09	1.4E-05	0.005	7.2E-03	5.2E-04
Ifn_Beta_Up	-2.92	5.4E-05	0.012	1.2E-02	1.3E-03
Sana_Ifng_Endothelial_Up	-2.88	6.6E-05	0.014	1.2E-02	1.7E-03
Ifn_Any_Up	-2.76	1.1E-04	0.019	2.4E-02	1.4E-03
Dac_Bladder_Up	-2.65	2.8E-04	0.036	2.4E-03	4.0E-02
Grandvaux_Ifn_Not_Irf3_Up	-2.76	2.8E-04	0.037	3.8E-02	2.6E-03
Ifna_Uv-Cmv_Common_Hc	-2.55	5.1E-04	0.056	1.6E-02	1.1E-02
Bennett_Sle_Up	-2.48	7.3E-04	0.071	1.4E-02	2.0E-02

Canonical Pathways	t-statistic	p-value	q-value	P.exp1	P.exp2

Tgf_Beta_Signaling_Pathway	3.15	2.2E-05	0.009	1.2E-03	1.3E-03
Wnt_Signaling	2.47	5.9E-04	0.099	3.2E-03	1.7E-02
Alkpathway	2.46	8.8E-04	0.11	9.8E-03	8.7E-03
Proliferation_Genes	2.27	1.3E-03	0.13	6.8E-03	1.9E-02
Cell_Proliferation	2.24	1.5E-03	0.15	2.1E-02	7.5E-03
Hematopoesis_Related_Trans	2.05	3.9E-03	0.31	1.8E-02	2.5E-02
Erythpathway	1.98	7.5E-03	0.46	2.7E-02	3.5E-02
Smooth_Muscle_Contraction	1.79	1.0E-02	0.54	2.7E-02	5.2E-02
Apoptosis	1.73	1.3E-02	0.60	7.1E-02	2.5E-02
Breast_Cancer_Estrogen_Signaling	1.61	2.0E-02	0.69	8.1E-02	3.6E-02

Top 10 most significantly differentially expressed experimental sets and canonical pathways were inferred by GAGE from human MSCs following an 8 hour BMP6 treatment. Two replicate experiments were done, each with BMP6 treated sample and control. Therefore GAGE was applied to each experiment and derived corresponding p-values (P.exp1–2). Gene sets were ranked based on global p-values from both experiments. FDR q-values were estimated to correct the global p-values for the multiple testing issue.

**Table 4:** Comparison between GAGE, PAGE and GSEA-g results from the BMP6-MSC dataset

Gene Sets & Methods		Top 10 abs(T/Z)	Top 10 p-values	Sign. Sets
Experiment Sets	GAGE	2.48	7.3E-04	39 (13)
	
	PAGE	24.6	1.3E-131	864 (940)
	
	GSEA-g	1.97	<1.0E-3	86 (77)

Canonical Pathways	GAGE	1.61	2.0E-2	7 (2)
	
	PAGE	10.9	8.8E-28	248 (297)
	
	GSEA-g	1.53	6.8E-2	6 (0)

The significantly enriched experimental sets and canonical pathways in human MSC following 8 hour BMP6 treatment were inferred by GAGE, PAGE and GSEA-g (permutation of gene labels). Top 10 t- (GAGE) or z- (PAGE) statistics or NES (GSEA) and p-values and the numbers of significant gene sets were shown with p-value ≤ 0.01 (or FDR q-value ≤ 0.10). Note that GSEA-g results shown were based on nominal p-values (or FDR q-values).

Using a p-value cutoff of <0.01, GAGE identified fewer gene sets than PAGE (Table 4). GAGE identified 39 significant experimental sets and 7 canonical pathways (Additional file 2). There were only 17 significant experimental sets and 4 canonical pathways (Additional file 2) after removing the redundancy among gene sets, which is reasonable number of pathways triggered by a single perturbation in a single cell line. In contrast, PAGE called 745 significant experimental sets and 187 significant canonical pathways. Most significant genes sets selected by PAGE were not significant according to GAGE using the same cutoff p-value (full result tables not shown). After removing the redundancy in these sets, there were more than 200 and 40 non-redundant experimental sets and canonical pathways respectively (not shown, Additional file 1: Supplementary Note 3). Presumably, PAGE made a large number of false positive calls. Similar differences between GAGE and PAGE were observed for the two lung cancer datasets and the type 2 diabetes dataset (Table 2 and Additional file 1: Supplementary Table 7). This difference came from the different statistical tests used by GAGE and PAGE, i.e. two-sample t-test vs one-sample z-test (detailed in the subsection of 'Dissection of major strategies employed by GAGE'). GSEA-g gave p-values and a predicted number of significant gene sets comparable to GAGE when nominal p-values were used (Table 4 and Additional file 1: Supplementary Table 9, full Table not shown).

Biologically, GAGE gene sets were mechanistically more relevant for BMP6 effects compared to those sets selected by PAGE. 9 out of 10 experimental sets inferred by GAGE (Table 3) are directly related to interferon or STAT pathway [30], which is a target of BMP signaling [31,32]. The experimental sets selected by PAGE alone have less connection to BMP (Additional file 1: Supplementary Table 8). GAGE and PAGE differed in 8 entries of the top 10 canonical pathways. Of GAGE predictions (Table 3), Wnt signaling [33,34], proliferation [35,36] are all known pathways or processes regulated by BMPs in MSC or osteoblastic cell lineages. BMPs regulate hematopoiesis and erythrocyte differentiation [37,38]. Breast cancer estrogen signaling interacts with BMP signal [39,40]. None of these pathways were significant according to PAGE (Additional file 1: Supplementary Table 8, full result table not shown). The GSEA-g top experimental sets overlapped with GAGE, but the canonical pathways were more similar to PAGE (Additional file 1: Supplementary Table 10).

Significant gene sets inferred by GAGE were consistent across replicate experiments and within the top 10 lists. The top 10 gene sets are almost the same if we used either one of the two experiments only (Table 3). The difference between the p-values from the two experiments almost never exceeded one order of magnitude. On the other hand, the top 10 gene set lists inferred by the PAGE and corresponding p-values are more different across the two experiments (Additional file 1: Supplementary Table 8, not all top sets for individual experiments included). There was also high level of internal consistency in the top 10 gene sets inferred by GAGE (Table 3). For example, 9 out of 10 experimental sets were directly related to interferon signal. Among the canonical pathways, there were two proliferation and two hematopoietic differentiation related pathways. In addition the high scoring Alk pathway overlapped with TGF beta and Wnt signaling pathways. In contrast, the PAGE and GSEA-g top gene sets had lower internal consistencies (Additional file 1: Supplementary Table 8–9). These results indicate that GAGE is a method robust against the heterogeneity in experiments or gene set definition. Notice that redundant gene sets representative of the same effect or pathway were kept here for exact comparison between methods, but they can be differentiated and combined by GAGE program if needed (Additional file 2).

### A microarray data based simulation study

We conducted simulation study to compare the performance of GAGE vs GSEA and PAGE in a more controllable setting. To minimize the potential artifact of using synthetic data, we used the type 2 diabetes dataset which has been analyzed in the first part of the Results. We chose this large clinical dataset so that all methods including the sample randomization based GSEA are applicable. Also, to make the simulation tractable for GSEA, we employed a sub-dataset with 2000 randomly sampled genes from the full set of 17000 genes. While the dataset is real microarray data, we synthesized the testing gene sets with controlled levels of differential expression (or degrees of enrichment in up- or down- regulated genes, details described in Methods). We then applied GAGE, PAGE or GSEA to score these testing gene sets, and evaluated whether the enrichment scores reasonably reflect the differential expression levels of these testing gene sets.

**Figure 2:**
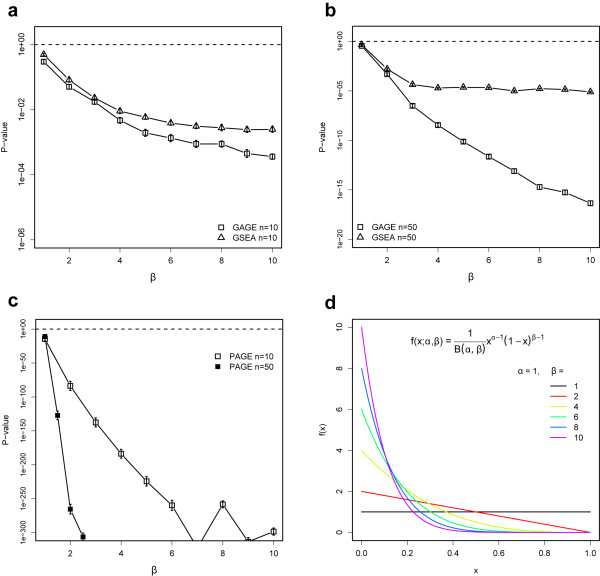
**A simulation study using microarray data and synthetic testing gene sets**. (a-c) p-values on the differential expression of testing gene sets with increasing levels of enrichment of up-regulated genes, when GAGE (a, b), GSEA (a, b) and PAGE (c) were applied. (d) The series of beta distribution curves with 1 ≤ β ≤ 10 and fixed α = 1 used to sample the testing gene sets with increasing levels of up-regulation from a sorted whole gene list. For each β value, we generated testing gene sets of two different size n = 10 genes (small sets) and n = 50 genes (large sets), 100 gene sets each. We then applied GAGE, PAGE or GSEA to test the overall expression level up-regulation in these gene sets. Mean p-values plus with standard error were shown. See Methods and Results for details. Note that GAGE with both 1-on-1 and 1-on-grp options produces similar results, although only the former is shown here.

Similar to the analysis results described above, while GAGE and GSEA gave more sensible p-values in the simulation, PAGE resulted in unrealistically small p-values on the order of 10^-324^. (Figure 2c). The fact that p-values started from 10^-11 ^(n = 10) or 10^-15 ^(n = 50) for gene sets with no up-regulation at all (β = α = 1) shows that PAGE suffers from low specificity. In other words, the extremely small p-values did not indicate high sensitivity but rather a high false positive rate for PAGE. On the other hand, GAGE and GSEA are selective and started from insignificant p-values for the negative control gene sets with β = α = 1. Compared to GSEA, GAGE gave smaller p-value for gene sets with different levels of up-regulation (Figure 2a–b). In other words, GAGE is more sensitive than GSEA. This improvement does not come at the cost of lower specificity (Additional file 1: Supplementary Figure 2a-b and detailed next). Note that GSEA reached sensitivity cap (around β = 7 for n = 10 and β = 4 for n = 50, Figure 2a–b). Out of all three methods, only GAGE produced strictly monotonically decreasing p-value curves that closely reflected the increasing up-regulation levels of the testing gene sets with increasing β.

We further compared the p-values inferred by these three methods under null condition by using testing gene sets with strictly no up-regulation (Additional file 1: Supplementary Figure 2). GAGE with 1-on-1 and grp-on-grp options and GAGE-r (details in Methods) derived null p-values closely following a uniform distribution. This further confirmed the theoretical soundness of GAGE, and that the improved sensitivity of GAGE (Figure 2a–b) compared to GSEA does not rely on a bias in the null distribution (Additional file 1: Supplementary Figure 2a-b). The null simulation indicates that both GAGE and GSEA are equally selective against false positive (Additional file 1: Supplementary Figure 2a-b). However, simulation with different levels of up-regulated gene sets shows GAGE is more sensitive to true positive (i.e. real difference) (Figure 2a–b). GAGE with the 1-on-grp option (details in Methods) derived null p-values that are slightly different from the uniform distribution, and is more likely to produce false positive results compared to GAGE with the default 1-on-1 option. However, the GAGE 1-on-grp comparison would still work reasonably well as a computationally fast option based on our results using experimental data (Table 5). In contrast, PAGE derived an extremely biased null p-value distribution. Over 40% of the p-values are essentially 0, another 40–50% are 1. Clearly, in consistent with all our earlier observations, PAGE produces a high false positive rate.

**Table 5:** The three comparison schemes of GAGE, 1-on-1, 1-on-grp and grp-on-grp

Gene Sets & Methods		Overlap	Top 10 p-values	Metastasis	Tumor	Sign. Sets
Experimental Sets	1-on-1	4	1.3E-28, 1.2–9	2, 3	5, 5	201 (254), 55 (47)
	
	1-on-grp	4	4.2E-35, 2.4E-13	3, 5	6, 7	242 (283), 120 (124)
	
	grp-on-grp	3	6.5E-8, 1.8E-4	3, 4	6, 8	52 (69), 17 (0)

Canonical Pathways	1-on-1	6	7.2E-5, 3.7E-03	9, 9	9, 9	18 (12), 8 (5)
	
	1-on-grp	5	6.7E-6, 7.5E-4	10, 9	10, 9	20 (16), 10 (8)
	
	grp-on-grp	0	1.1E-1, 6.1E-2	4, 5	6, 5	0 (0), 0 (0)

Top 10 significantly enriched experimental sets and canonical pathways in poor clinical outcomes vs good outcomes were inferred by GAGE using these three different comparison schemes from two published lung adenocarcinoma data sets [3]. Data columns are overlap between top 10 gene sets for the two studies, top 10 p-values, number of top 10 gene sets related to metastasis (bt) and tumor (t and bt), and numbers of significant gene sets with p-value ≤ 0.001 (or with FDR q-value ≤ 0.01).

### Impact of GAGE strategies: gene set separation, two-sample t-test, and one-on-one comparisons

Compared to PAGE and GSEA, GAGE employs three different strategies: 1) gene set separation, 2) two-sample t-test, and 3) one-on-one comparisons between experiment and control samples. In this section, we show the impact of each of these three strategies in representative analyses, although these strategies have been consistently effective when applied to multiple datasets covered or not covered in this paper. We compare GAGE to PAGE on these aspects if possible, or to GAGE variants which ensembles PAGE in each one of these three aspects for exact comparison. GSEA is either not or less comparable in these aspects.

#### Gene set separation

**Figure 3:**
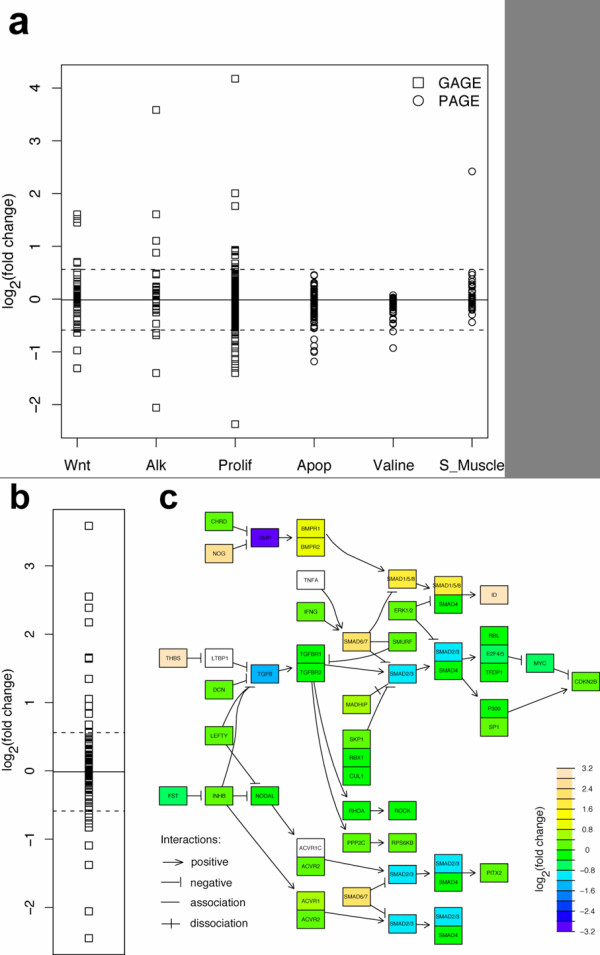
**GAGE captured canonical pathways which are significantly perturbed towards both directions following 8 h BMP6 treatment in human MSC**. (a) Gene expression level changes in the top 3 different significant canonical pathways inferred by GAGE and PAGE. (b) Gene expression level changes in the canonical TGF beta signaling pathway and (c) plotted in pseudo-color on the pathway topology derived from KEGG database. The solid horizontal line and dashed lines in (a-b) mark the mean fold changes of all genes and the positive/negative two times standard deviation from the mean respectively. Note that in (c), one KEGG node may correspond to multiple closely related genes with the same function, and the maximum fold changes among these genes are plotted as the color of the node.

In contrast to PAGE and GSEA, GAGE separates canonical pathways from experimental sets and considers potential perturbations in both directions (i.e. up and down regulation simultaneously) in canonical pathways. Expression data directly showed that genes in the most relevant canonical pathways are regulated in both directions (Figure 3). Figure 3a shows the gene expression level changes following BMP6 treatment in top 3 different significant canonical pathways inferred by GAGE and PAGE (Table 3 and Additional file 1: Supplementary Table 8). These canonical pathways inferred by GAGE are directly related to BMP induced osteoblast differentiation [34,35] (Alk pathway is essentially TGF Beta signaling + Wnt signaling). Figure 3b shows the gene expression level changes in the TGF beta-BMP signaling pathway following BMP6 treatment. This pathway is a presumable gold standard as it is the primary signal triggered directly by BMPs (KEGG). The changes of gene expression are not uniform. The TGF-beta pathway includes both positive effectors such as BMPs, BMPR1–2, SMAD1/5/8, ID1–4, and THBS, and negative effectors such as NOG, SMAD2/3, and SMAD6/7. Clearly, both types of effectors were regulated up and down. Genes are regulated in both directions not only for the whole pathway but also within the sub-pathways like BMP or TGF-beta signaling branches. These results demonstrate that genes in canonical pathways are frequently up- and down-regulated simultaneously because 1) they play positive or negative roles [20] and 2) homeostatic mechanisms tend to bring a certain level of balance back to the system when it is perturbed [19]. Therefore, it is necessary to treat canonical pathways differently from experimental sets and count both up and down regulation when doing gene set analyses.

Compared to the top 10 canonical pathways assuming one-way changes, the top 10 canonical pathways allowing two-way changes better described BMP induced osteoblast differentiation mechanistically (Table 3 and Additional file 1: Supplementary Table 11). TGF beta signaling, Wnt signaling and cell proliferation are all known essential signals or processes for osteoblast differentiation [34,35], yet they are not significant in the one-way changing list (Additional file 1: Supplementary Table 11, full Table not shown). One-way assumption tends to select metabolism pathways (6 out of 10 canonical pathways in Additional file 1: Supplementary Table 11), which are likely to be tightly coregulated as relative simple functional group. In other words, top canonical pathways with one-way changes are still interesting if they are not complicated regulatory pathways.

#### Two-sample t-test

**Figure 4:**
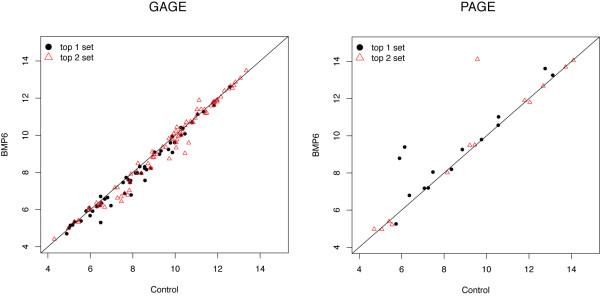
**Differential gene expression in the top 2 significant experimental sets inferred by GAGE or PAGE**. Gene expression levels are log 2 based, and compared between human MSC with 8 hour BMP6 treatment vs control. Results for the first experiment are shown, and the second replicate experiment is similar.

**Figure 5:**
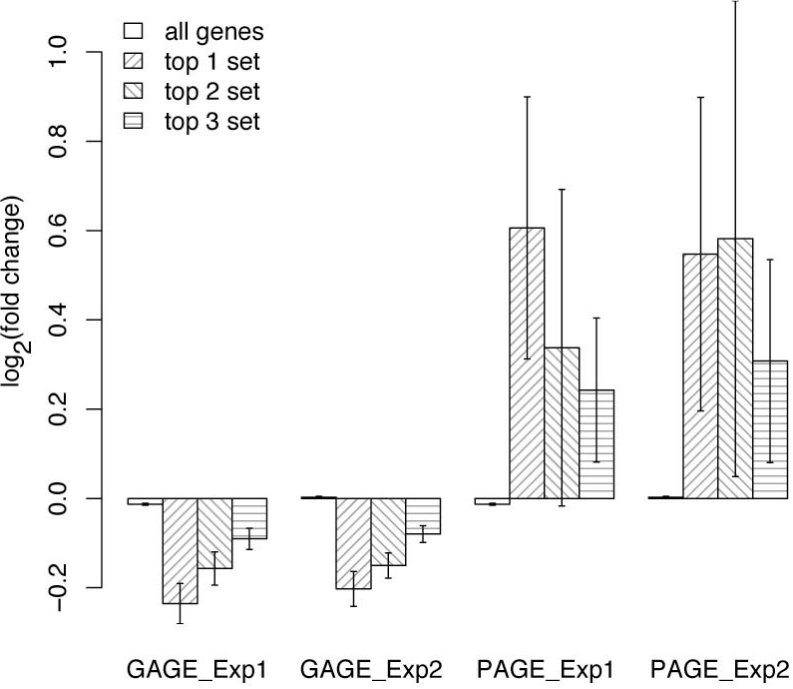
**Gene expression fold changes (log 2 based) in the top 3 significant experimental sets inferred by GAGE or PAGE**. For each gene set, the bar height represents mean and error bar represent standard error of gene expression fold changes induced by 8 hour BMP6 treatment in human MSC. GAGE uses two-sample t-test and PAGE does one-sample z-test. PAGE frequently selected gene sets with extreme up or down regulation in a few genes and almost no changes in the rest. Such gene sets have too large within-group variances to be called significantly different from the background based on two-sample t-test, even though their mean fold changes are big.

GAGE uses a two-sample t-test to compare expression level changes of a gene sets to the whole set background, whereas PAGE uses a one-sample z-test. GAGE's use of a two-sample t-test has three effects. First, two-sample t-test considers the variance for both the target gene set distribution as well as the background distribution (Formula 1), while a one-sample z-test only considers the variance for the background distribution and ignores the effect of specific target gene set distribution (Formula 2). The background variance is small and often negligible compared to the within gene set variance, hence PAGE can produce unrealistically large z-scores and small p-values (Additional file 1: Supplementary Table 8) in contrast to GAGE (Table 3). Second, the two-sample t-test used by GAGE identifies gene sets with modest but consistent changes in gene expression level, whereas PAGE tends to identify gene sets with a few extremely changed outliers (Figure 4, more comments in Additional file 1: Supplementary Note 4). In other words, GAGE is more robust to experimental noise or variations in gene set definitions than PAGE. Many top gene sets selected by PAGE were not significant according to GAGE (Table 3, Additional file 1: Supplementary Table 8, full tables not shown) because the within gene set variance is too large (Figure 5). On the other hand, significant gene sets inferred by GAGE are almost always selected as significant by PAGE (Table 3, Additional file 1: Supplementary Table 8, full tables not shown). Said another way, GAGE is as sensitive (high true positive calls) as PAGE, but more specific (low false positive calls) than PAGE (also see Additional file 1: Supplementary Figure 2a-b). Third, there is higher level of consistency within the top 10 gene sets inferred by GAGE (Table 3) than by PAGE (Additional file 1: Supplementary Table 8), and between the top 10 gene sets across experiments (Table 3 vs Additional file 1: Supplementary Table 8). This consistency is because the two-sample t-test is more robust than one-sample z-test for gene set analysis. All these observations for PAGE also apply to GAGE-z (GAGE variant doing one-sample z-test, Additional file 1: Supplementary Table 12).

With the classical two-sample t-test as the default of GAGE, we also looked at a rank-based two-sample t-test as an alternative (GAGE-r), which is potentially less sensitive to the violation of normal distribution assumption and expression outliers. GAGE-r gave similar results (Additional file 1: Supplementary Table 13) in terms of the significant gene set list, the number of significant gene sets (not shown), p-values and q-values. This result further confirmed the robustness of GAGE method and validated two-sample t-test as the default option.

#### One-on-one comparisons

GAGE carries out one-on-one comparisons between experiments and controls, whereas PAGE compares experiments and controls as two groups together. One-on-one comparisons are natural when the experiment samples and controls are paired. This one-on-one pairing is still preferred over group-on-group comparison even though experiments are not pair-matched for two reasons. First, multiple tests on all experiment-control pairs are more statistically powerful than single test on group averages, as the p-values (hence FDR q-values) would be orders of magnitude smaller for the one-on-one comparisons versus the group comparisons (Table 5, and Table 3 vs Additional file 1: Supplementary Table 14). Second, comparisons between two specific samples makes sense but not between two sample groups when the net effect of the whole gene set is non-additive, for instance, being expressed as mean of the absolute fold changes for canonical pathways (Additional file 1: Supplementary Note 5). As expected, a one-on-one comparison approach produced more consistent and biologically meaningful results across independent studies (Table 5). The enumeration of all one-on-one comparisons is not always advantageous as it can be relative slow for datasets with large number of replicates. To speed up the analysis of larger datasets, we can take the average gene expression levels for all controls as a single reference state and do gene set analysis on each experiment sample vs this reference state, because controls are often more homogenous than experiments. Correspondingly, GAGE has the options for three-way comparison schemes specified as 1-on-1, 1-on-grp and grp-on-grp. The option 1-on-grp produces similar results to 1-on-1 but different results to grp-on-grp (Table 5 and Additional file 1: Supplementary Table 15). The difference between these three options is better shown when the sample conditions are complicated as in the large clinical datasets above.

## Discussion

In this work we have presented a new gene set analysis method GAGE that is generally applicable to gene expression datasets of all sample sizes and experimental designs and in general performs better than two most frequently used methods. We have demonstrated GAGE's performance by comparing it to GSEA and PAGE extensively in the following three aspects: (1) consistency across parallel studies or experiments; (2) sensitivity and specificity of the pathway inference; (3) biological relevance of the pathways identified.

Our results show a significant impact of separating gene sets into pathway and experimentally derived gene sets as is shown in Figure 3. We showed that two-way perturbations commonly occur in regulatory pathways (Figure 3 and Table 1, also in Table 3 and Additional file 1: Supplementary Table 6), which would otherwise be overlooked (Additional file 1: Supplementary Table 11). However, pathway derived gene sets do not always show regulation in both directions. For example, we see that metabolic pathways or functional groups such as GO term categories tend to be coregulated toward one direction (Additional file 1: Supplementary Table 11). Strictly speaking, many of these gene sets are not signaling pathways and could be further separated from canonical signaling pathways (such as in MSigDB collection c2). In response to this observation, GAGE provides the option for two rounds of screening on MSigDB pathway sets. The first round assumes two-way regulation for regulatory signaling pathways while the second round assumes one-way for coregulated functional groups.

GAGE made two assumptions in conducting two sample t-tests on the log based fold changes of target gene set and control sets. The first assumption is approximate normal distribution for the mean fold change of the two sets. The central limit theorem states that the distribution of an average of sampled observations is normal regardless of the nature of parent distribution when sampling size is large enough. Indeed, the mean of fold change values for gene sets with ≥ 10 genes are close to normal distribution as shown by q-q plot previously [5]. The second assumption is that the fold changes of genes are independent and identically distributed (IID). Dependency between genes has been a concern for all gene randomization methods [11]. However, Netwon et al [6] argued that dependency is not necessarily an issue when GSA was conditioned on the differential expression analysis results (like fold changes). Moreover, we think dependency (coregulation) is rare for randomly sampled control gene sets. For most curated gene sets there is no coregulation under the specific condition of the microarray study (even though they might be under certain other condition), and the null hypothesis holds. For the few interesting gene sets where genes are coregulated, there will be a significant difference in expression between these sets and random control sets, hence the null hypothesis gets rejected. Therefore, gene sets which violate the IID assumption are the few significant sets and will be captured this way [5,6]. GAGE results clearly showed that our arguments work. The same logic has also been quite successful in well established gene randomization methods [5-7].

The one-on-one comparison scheme is generally applicable to datasets of all sample sizes and experiment designs. We used a meta-test to infer a global p-value for all the individual comparison p-values. The global p-values and the number of significant gene sets we derived are sensible. As in common statistical tests, these p-values tend to decrease when the sample size increases, and can become small for large datasets like the lung cancer datasets (Table 1), hence the number of significant gene sets can be large especially when all the redundant gene sets are kept (Table 2 and Additional file 1: Supplementary Table 7). This result is still sensible because large clinical datasets (like the lung cancer studies) are generally more heterogeneous than small experimental datasets (like the BMP6 study).

There are frequently multiple significant gene sets that share multiple genes or represent the same regulatory mechanism, especially for experimental gene sets. This redundant gene sets problem has been discussed elsewhere in detail [41]. In response to this issue, GAGE has the option to combine redundant gene sets and give more concise significant gene set lists (Additional file 2). In this work, we chose not to combine these redundant gene sets for exact comparison between methods. As a benefit of not merging these sets, we took these overlapping sets as an internal control to validate the internal consistency of the predictions.

There is also a multiple testing issue, i.e. gene sets may become significant when the gene set number is large. Classical FDR procedures like Benjamini-Hochberg (BH) [42] and Bonferroni [43] corrections tends to be conservative. Such adjustment is further complicated when gene sets contain different numbers of genes (not exactly the same null hypothesis test for different gene sets). Hence gene randomization based GSA methods like PAGE [5] and T-Profiler [7] do not consider this adjustment (we added the FDR procedure to PAGE in Additional file 1: Supplementary Table 1, 6 and 8 for comparison purpose). Sample randomization based GSA methods like GSEA suffer from conflicting ordering between FDR q-values and nominal p-values (Additional file 1: Supplementary Table 2, 6 and 9). In GAGE, the one-on-one comparison and one-on-group comparison schemes not only gives us more testing power and robustness, but also provides the framework to conduct a unified and rigorous FDR procedure for gene sets of different sizes. Because the meta-test on *K *p-values (Formula 5, see methods for details) is the same (with the same null hypothesis) for all gene sets despite of their different size.

## Conclusion

In this work, we present a novel method GAGE for gene set analysis (GSA). GAGE is generally applicable to gene expression datasets with different sample sizes and experimental designs, hence greatly expands the applicability of GSA. In both simulation experiments and multiple microarray data analyses, GAGE consistently outperformed two most frequently used GSA methods, GSEA and PAGE in three major aspects: (1) consistency across repeated studies/experiments; (2) sensitivity and specificity; (3) biological relevance of the regulatory mechanisms inferred. GAGE reveals novel and relevant regulatory mechanisms from both published and previously unpublished microarray studies.

## Methods

A schematic overview of GAGE procedure is shown in Figure 1. Here we describe the major steps of GAGE.

### Gene sets separation

GAGE uses curated gene sets [3] collected from individual studies or pathway databases for regulatory mechanisms inference. In contrast to other gene set analysis approaches, GAGE requires that each curated gene set be identified as either a pathway set (canonical pathways) or an experimentally derived differential expression set (experiment sets). GAGE treats these two categories differently. Genes in an experimental set are assumed to be regulated in the same direction, either all up or all down, as they were in the original study. In contrast, genes associated with a pathway gene set may be heterogeneously regulated in either direction. This separation better reflects the origin of the gene set and is therefore expected to produce better results.

For an experimental set the test statistic (score) used in GAGE is the average of the per-gene test statistics–similar to the scoring scheme used by other gene set analysis methods. However, for canonical pathways GAGE uses the average of the absolute values of the per gene test statistics to account for both up- and down-regulation.

### Significance test

To test whether a gene set is significantly correlated with a phenotype or an experiment condition, we exam the fold changes of gene expression level in the experiment condition (or phenotype) vs control condition. Correspondingly, we want to test whether the mean fold changes of a target gene set is significantly different from that of the background set (the whole gene set of the microarray). This is a prototype two-sample t-test, as shown in Formula 1, in contrast to the one-sample z-test used in PAGE [5] shown in Formula 2.

(1)

(2)

Where m, s and n are the mean fold change (log ratio of expression levels), standard deviation, and number of genes in a particular gene set, and M and S are the mean fold change and standard deviation for all of the genes in the dataset. Notice that this is a two sample t-test between the interesting gene set containing n genes and a virtual random set of the same size derived from the background (comparable to the one-sample z-test control set in Formula 2). Two sample t-test would be inaccurate when the two sample sizes are not comparable [44]. The degree of freedom (df) for this two-sample t-test (Formula 1) with unequal variance is given in Formula 3. The common range for df would be n-1 (when s>>S) to 2n-2 (when s = S). Actually df has little effect on the p-values when n is large enough (for most gene sets), where t-distribution is nearly normal. The assumptions we made for the two-sample t-test are described in the Discussion section in detail.

(3)

With the classical two-sample t-test as the default of GAGE, we also implement a rank-based two-sample t-test [45] as an alternative (GAGE-r). This rank based t-test is equivalent to the non-parametric Wilcoxon Mann-Whitney test [45]. These rank based alternatives do not assume normal distribution of the samples and are potentially less prone to outliers compared to the classical parametric two-sample t-test. To conduct the rank test, we transform the data to ranks and then performing the two-sample t-test on the transformed data.

#### One-on-one comparison between microarray experiment and control samples

For microarray studies with one-on-one paired experiment and control samples, we calculate fold changes and carried out gene set significance tests for each experiment versus control sample pair. For microarray studies with multiple unpaired experimental and control samples, GAGE has two options: 1-on-1 and 1-on-grp. In 1-on-1 we enumerate all pairs of experiment-control and do gene set significance tests. In the 1-on-grp option we take the average gene expression level for all control samples as the sole reference, compare each experimental sample against this reference and do gene set significance tests. 1-on-1 is more rigorous theoretically. Our experiment showed that 1-on-grp gives nearly identical results and is much faster when the sample size is large. We take 1-on-1 as our standard, and leave 1-on-grp as a computationally fast option (default for unpaired experiments in this paper). We also implemented the commonly used comparison between experiment group and control group as the grp-on-grp option.

#### Combination of multiple comparisons or experiments

GAGE derived multiple t-statistics and p-values from Formula 1 when doing 1-on-1 or 1-on-grp comparison for datasets with replicate samples. We derive a global p-value by combining these individual p-values. Individual p-value follows a Uniform(0,1) distribution under the null hypothesis of the two-sample t-test and the negative log sum of *K *independent p-values follows a Gamma(*K*,1) distribution. Hence we can do a meta-test for all the p-values of a gene set across multiple samples (Formula 4–5).

(4)

(5)

Note that this analysis assumes that individual p-values come from independent comparisons. However, the 1-on-1 comparisons are not all independent for unpaired studies (with *k *= 1,., *K *experiments and *l *= 1,., *L *controls), thus we need to take the average of the p-values for all *L *comparisons of a experiment to different controls as the p-value for that experiment (Formula 6) and then apply Formula 5 to these *K *independent p-values.

(6)

To correct the p-values for the multiple testing issue, we estimate FDR by using fdrtool [46], a unified approach recently established. Compared to the traditional FDR procedures, fdrtool estimates FDR based on the empirical null distribution, hence allow more realistic and less conservative correction of p-values [46]. In rare cases, fdrtool may perform less ideal likely due to the extreme distribution of input p-values. We provide the classical Benjamini-Hochberg (BH) [42] procedure as a backup option.

### Implementation of GAGE

GAGE is implemented in the statistical computing language R and is freely available online [47]. The gene sets used in this paper are from the Molecular Signature Database of GSEA website [23]. From this site, we used the curated gene sets (collection c2), and treat the two sub-collections experimental sets (CGP: chemical and genetic perturbations) and canonical pathways differently. There are 16966 unique gene symbols in c2, 3834 of them are nonstandard. Among these nonstandard symbols, 1190 were converted standard symbols automatically by using GAIQ database [48]. Database access and scripts for the gene symbol standardization is available upon request.

### Comparison software

GAGE was compared to two widely used gene set analysis software packages: PAGE and GSEA. GSEA-P-R.1.0 was downloaded form GSEA website [49], and PAGE is implemented in R as part of GAGE package based on description of the authors [5] and source codes in PGSEA package [50].

### Datasets

The gene set analysis software was compared using three datasets including two large studies and one small one.

The two large studies included a lung cancer set was provided with GSEA-R package [49] and a type 2 diabetes dataset comes from ChipperDB [51]. These datasets were chosen because they were originally used to validate and/or compare GSEA [3,4] and PAGE [5]

The small dataset is a gene expression study from our group describing human MSC response to 8 hours of exposure to the signaling molecule BMP6. This dataset includes two experimental groups each with paired treatment and control samples, resulting in a total of 4 gene chips. We have deposited the dataset into Gene Expression Omnibus (GEO) repository (accession number GSE13604). For the use in this paper, the raw data were processed by using RMA implemented in the Bioconductor Affy package [52] with up-to-date probe set definition (.CDF file) based on Entrez Gene sequence, Hs133P_Hs_ENTREZG_8 [53]. Annotation data were retrieved from the GAIQ website [48]. The type 2 diabetes dataset was processed similarly from raw data files.

### Synthesize the testing gene sets with controlled levels of differential expression

While the dataset for simulations study is real microarray data, we synthesized the testing gene sets with controlled levels of differential expression (or degrees of enrichment). We ranked all genes based on average fold change between the two sample groups (i.e. type 2 diabetes samples and controls) from most up-regulated to most down-regulated. We then sampled gene sets following a series of different Beta-distributions in gene ranks. One of the two parameters, α is fixed to 1, and the other parameters β takes values from integer 1 to 10 (Figure 2d), which control the shape of Beta distribution (Figure 2d) hence the degree of enrichment of the up-regulated genes (or the level of up-regulation of the gene set): the uniform distribution at β = 1 corresponds to no enrichment at all and the highly skewed distribution at β = 10 corresponds to highest enrichment of up-regulated genes (Figure 2d). For each β value, we generated 100 testing gene sets of 10 genes (small sets) and 100 sets of 50 genes (large sets). We then applied GAGE, PAGE or GSEA to score these testing gene sets, and evaluated whether the enrichment scores reason ably reflect the differential expression levels of these testing gene sets. Note that α and β are symmetric parameters. When we exchange them, the simulation remains the same except that the gene sets were enriched with down-regulated genes.

## Authors' contributions

WL and PJW conceived and designed the study; WL and KS designed the statistical procedure; WL conducted the research and wrote the computer program; MSF and KDH conducted the BMP6-MSC microarray experiment. WL, MSF, KS, KDH and PJW drafted the manuscript. All authors read and approved the final manuscript.

## Supplementary Material

Additional File 1 **Supplementary tables, figures and notes.** Click here for file

Additional File 2 **Full and non-redundant lists of significant gene sets inferred by GAGE when applied to the BMP6-MSC dataset.** Click here for file
