# Supplementary material for: GAGE: generally applicable gene set enrichment for pathway analysis
Source: BMC Bioinformatics. 2009 May 27;10:161. doi: 10.1186/1471-2105-10-161 (PMC2696452; doi:10.1186/1471-2105-10-161)
Supplement: Additional File 1 — Supplementary tables, figures and notes. [file 1471-2105-10-161-S1.pdf]

Supplementary Tables

Boston study				Michigan study			
Experimental Sets	p-val	q-val	Notes	Experimental Sets	p-val	q-val	Notes
**Serum_Fibroblast_Cel**	0	0	bt,c	**Serum_Fibroblast_Cell**	9.E-178	4.E-175	bt,c
Brca_Er_Neg	2.E-273	3.E-271	bt	**Zhan_Mm_Cd138_Pr_**	1.E-124	3.E-122	bt
Rcc_Nl_Up	5.E-266	5.E-264	t	**Tarte_Plasma_Blastic**	4.E-104	6.E-102	c
**Tarte_Plasma_Blastic**	3.E-218	2.E-216	c	Yu_Cmyc_Dn	7.E-94	6.E-91	t
Vhl_Normal_Up	2.E-204	1.E-202		Et743_Sarcoma_Dn	1.E-92	1.E-90	t
**Zhan_Mm_Cd138_Pr**	3.E-195	2.E-193	bt	Dox_Resist_Gastric_Up	8.E-91	7.E-89	bt
**Lei_Myb_Regulated_**	2.E-189	9.E-188	bt,c	Greenbaum_E2a_Up	1.E-90	8.E-89	
Cancer_Undifferentiate	2.E-183	1.E-181	bt	Idx_Tsa_Up_Cluster3	3.E-90	2.E-88	c
Hcc_Survival_Good_Vs	2.E-173	8.E-172	bt	Lee_Tcells3_Up	7.E-88	4.E-86	
Serum_Fibroblast_Core	1.E-170	3.E-169	bt	**Lei_Myb_Regulated_G**	2.E-85	1.E-83	bt,c
Canonical Pathways	p-val	q-val	Notes	Canonical Pathways	p-val	q-val	Notes
**Pyrimidine_Metabolis**	2.7E-81	4.7E-79		**Breast_Ductal_Carcin**	3.2E-49	5.7E-47	bt
Dna_Replication_React	1.9E-68	1.6E-66	c	Breast_Cancer_Estrogen	2.3E-39	2.1E-37	bt
Cell_Cycle	1.6E-62	9.0E-61	c	Ctlpathway	1.2E-37	3.3E-35	
**Cell_Cycle_Kegg**	1.4E-58	5.9E-57	c	Tcrapathway	9.1E-34	1.3E-31	
**Breast_Ductal_Carcin**	3.7E-50	1.3E-48	bt	Thelperpathway	2.4E-31	2.2E-29	
G1_To_S_Cell_Cycle_	5.6E-43	1.6E-41	c	**Cell_Cycle_Kegg**	3.7E-30	2.2E-28	c
**Pgc**	1.4E-29	3.4E-28		**Pgc**	2.5E-29	1.1E-27	
Ubiquitin_Mediated_Pr	2.5E-28	5.4E-27	t	Glycolysis_And_Glucon	1.6E-28	5.7E-27	c
Proteasome_Degradatio	1.1E-27	2.0E-26	t	**Pyrimidine_Metabolis**	5.9E-28	1.7E-26	
Gpcrdb_Class_A_Rhod	4.2E-26	1.2E-23	bt	Cskpathway	3.7E-27	2.1E-25	bt

Supplementary Table 1. PAGE applied to the two lung cancer datasets of large sample sizes. Top 10 most significantly enriched experimental sets and canonical pathways in poor clinical outcomes vs good outcomes were inferred by PAGE from two published lung adenocarcinoma data sets used in GSEA paper [1]. Both positively and negatively regulated gene sets were collected and ranking by the p-value, and by absolute value of z statistics (not shown) for ties. Experiment and results description here is the same as Table 1. Note that PAGE originally included no p-value adjustment for the multiple testing issue, we added FDR procedure to PAGE here for comparison purpose.

Boston study				Michigan study			
Experimental Sets	p-val	q-val	Notes	Experimental Sets	p-val	q-val	Notes
Egf_Hdmec_Up	3.9E-03	2.4E-01	c	Tnfalpha_30min_Up	4.2E-03	1.3E-01	c
Bcnu_Glioma_Nomgmt	2.1E-03	2.6E-01		Hif1_Targets	2.2E-03	1.4E-01	t,c
Tsadac_Hypometh_Ovc	2.0E-03	2.7E-01	t	Bhattacharya_Esc_Up	6.4E-03	1.6E-01	c
Uvb_Nhek3_C2	4.1E-03	2.8E-01	t	Mense_Hypoxia_Up	<1E-03	1.6E-01	c
**Tgfbeta_C1_Up**	5.7E-03	3.1E-01		**Tgfbeta_C1_Up**	2.3E-03	1.7E-01	
Rome_Insulin_2f_Up	2.0E-03	3.1E-01		5fu_Resist_Gastric_Dn	6.2E-03	2.2E-01	bt
Smith_Htert_Up	<1E-03	3.2E-01	t,c	Xu_Atra_Plusnsc_Dn	2.1E-03	2.4E-01	bt
Zucchi_Epithelial_Up	3.9E-03	3.3E-01	t	Hypoxia_Reg_Up	<1E-03	2.4E-01	c
Kenny_Wnt_Up	1.9E-03	3.3E-01	t	Coller_Myc_Up	6.4E-03	2.7E-01	c
Hypoxia_Review	<1E-03	5.8E-01	bt,c	Shipp_Fl_Vs_Dlbcl_Dn	1.1E-02	2.8E-01	t
Canonical Pathways	p-val	q-val	Notes	Canonical Pathways	p-val	q-val	Notes
P53hypoxiapathway	<1E-03	6.2E-02	t,c	Badpathway	<1E-03	1.5E-02	t
Aminoacyl_Trna_Biosy	5.9E-03	2.4E-01		Cskpathway	1.9E-03	2.0E-02	bt
Trna_Synthetases	1.0E-02	2.7E-01		Amipathway	1.9E-03	2.0E-02	
Nucleotide_Metabolism	1.8E-02	2.9E-01		Gluconeogenesis	<1E-03	1.1E-01	c
Cdc42racpathway	1.1E-02	5.2E-01	bt	Glycolysis	<1E-03	1.1E-01	c
Proteasome	5.3E-02	5.3E-01	t	Il12pathway	1.4E-02	1.1E-01	t
Dna_Replication_React	7.5E-02	5.3E-01	c	Tcrapathway	7.7E-03	1.3E-01	t
Proteasomepathway	5.2E-02	5.4E-01	t	Go_Ros	4.0E-03	1.4E-01	t
Proteasome_Degradatio	7.9E-02	5.6E-01	t	Vegfpathway	4.4E-03	1.6E-01	c
Pyrimidine_Metabolism	6.9E-02	5.8E-01		Glycolysis_And_Glucon	6.5E-03	1.7E-01	c

Supplementary Table 2. GSEA applied to the two lung cancer datasets of large sample sizes. Top 10 most significantly enriched experimental sets and canonical pathways in poor clinical outcomes vs good outcomes were inferred by by GSEA from two published lung adenocarcinoma data sets used in GSEA paper [1]. Both positively and negatively regulated gene sets were collected and ranking by the FDR q-value, and the nominal p-values for ties. Experiment and results description here is the same as Table 1.

Boston

Gene Sets & Methods		GAGE	PAGE	GSEA
Experiment Sets	GAGE	NA	5	0
	PAGE	5	NA	0
	GSEA	0	0	NA
Canonical Pathways	GAGE	NA	1	0
	PAGE	1	NA	3
	GSEA	0	3	NA

Michigan

Gene Sets & Methods		GAGE	PAGE	GSEA
Experiment Sets	GAGE	NA	5	0
	PAGE	5	NA	0
	GSEA	0	0	NA
Canonical Pathways	GAGE	NA	0	0
	PAGE	0	NA	3
	GSEA	0	3	NA

Supplementary Table 3. Overlaps between GAGE, PAGE and GSEA results from the two lung cancer datasets. The top 10 most significantly enriched experimental sets and canonical pathways in poor clinic outcomes vs good outcomes were inferred by GAGE, PAGE and GSEA from two published lung adenocarcinoma data sets used in the GSEA paper [1].

Boston study				Michigan study			
Experimental Sets	p-val	q-val	Notes	Experimental Sets	p-val	q-val	Notes
**Uvb_Nhek3_All**	1.0E-54	6.5E-52	t	**Tarte_Plasma_Blastic**	2.2E-29	2.0E-26	c
Peng_Glutamine_Dn	5.7E-54	1.8E-51	c	Cancer_Undifferentiated	7.5E-17	3.5E-14	bt
Lei_Myb_Regulated_G	6.1E-48	1.3E-45	bt,c	Caries_Pulp_Up	2.2E-15	2.2E-12	
**Tarte_Plasma_Blastic**	2.6E-46	4.1E-44	c	**Brca_Er_Neg**	2.3E-13	7.2E-11	bt
**Flechner_Kidney_Tra**	1.3E-44	1.7E-42		Serum_Fibroblast_Cellc	6.1E-13	1.4E-10	bt,c
Peng_Rapamycin_Dn	1.4E-39	1.5E-37	c	Li_Fetal_Vs_Wt_Kidne	1.1E-10	2.1E-08	t
Peng_Leucine_Dn	7.5E-37	6.7E-35	c	**Flechner_Kidney_Tran**	2.1E-10	8.0E-08	
**Brca_Er_Neg**	2.5E-31	2.0E-29	bt	Ageing_Kidney_Up	2.6E-10	8.7E-08	
Rcc_Nl_Up	5.3E-31	3.7E-29	t	Tarte_Mature_Pc	8.4E-10	2.0E-07	c
Cancer_Neoplastic_Met	1.3E-28	8.3E-27	t	**Uvb_Nhek3_All**	1.2E-09	1.5E-07	t
Canonical Pathways	p-val	q-val	Notes	Canonical Pathways	p-val	q-val	Notes
**Gpcrs_Class_A_Rhod**	8.0E-13	2.7E-10	bt	**Gpcrs_Class_A_Rhod**	2.8E-07	9.3E-05	bt
**Gpcrdb_Class_A_Rho**	6.8E-12	1.1E-09	bt	**Gpcrdb_Class_A_Rho**	1.5E-06	2.5E-04	bt
Fibrinolysispathway	2.7E-08	2.8E-06	bt	Androgen_Genes	4.1E-06	4.5E-04	bt
**Intrinsicpathway**	4.4E-08	3.7E-06	bt	**Intrinsicpathway**	7.4E-05	6.2E-03	bt
**Blood_Clotting_Casca**	4.5E-08	3.8E-06	bt	Cytokinepathway	1.1E-04	9.3E-03	bt
Tyrosine_Metabolism	2.4E-07	2.0E-05	bt	**Prostaglandin_And_Le**	6.6E-04	5.3E-02	bt
**Peptide_Gpcrs**	1.4E-06	1.2E-04	bt	Proliferation_Genes	7.1E-04	5.7E-02	c
**Prostaglandin_And_L**	3.6E-05	3.0E-03	bt	**Blood_Clotting_Cascad**	9.8E-04	7.6E-02	bt
Lairpathway	5.4E-05	4.5E-03		**Peptide_Gpcrs**	3.5E-03	2.3E-01	bt
**Extrinsicpathway**	7.2E-05	6.0E-03	bt	**Extrinsicpathway**	3.7E-03	2.4E-01	bt

Supplementary Table 4. GAGE applied to the two lung cancer datasets of large sample sizes following a 1-on-1 comparison scheme. Top 10 most significantly enriched experimental sets and canonical pathways in poor clinical outcomes vs good outcomes were inferred by GAGE from two published lung adenocarcinoma data sets used in GSEA paper [1]. Both positively and negatively regulated gene sets were collected and ranking by the p-value, and by absolute value of average t statistics (not shown) for ties. Experiment and results description here are the same as Table 1, except that poor clinic outcomes are compare to good outcomes following 1-on-1 comparison scheme rather than 1-on-grp.

Boston study				Michigan study			
Experimental Sets	p-val	q-val	Notes	Experimental Sets	p-val	q-val	Notes
**Tarte_Plasma_Blastic**	8.2E-13	2.3E-10	c	**Tarte_Plasma_Blastic**	4.6E-08	2.1E-05	c
**Uvb_Nhek3_All**	9.9E-10	1.1E-07	t	**Uvb_Nhek3_All**	4.1E-07	9.3E-05	t
Brca_Er_Neg	1.4E-09	1.3E-07	bt	Serum_Fibroblast_Cell	1.4E-06	2.1E-04	bt,c
Hcc_Survival_Good_Vs	1.9E-08	8.8E-07	bt	Cancer_Undifferentiated	4.5E-06	5.2E-04	bt
Cmv_Ie86_Up	2.6E-08	1.0E-06	c	Li_Fetal_Vs_Wt_Kidne	2.1E-05	1.9E-03	t
Peng_Leucine_Dn	2.9E-08	1.0E-06	c	**Lei_Myb_Regulated_G**	4.1E-05	3.0E-03	bt,c
Peng_Glutamine_Dn	2.9E-08	1.1E-06	c	Idx_Tsa_Up_Cluster3	6.5E-05	3.8E-03	c
Rcc_Nl_Up	3.4E-08	1.1E-06	t	Dox_Resist_Gastric_Up	7.8E-05	4.1E-03	bt
**Lei_Myb_Regulated_**	3.7E-08	1.1E-06	bt,c	Et743_Sarcoma_72hrs_	8.5E-05	4.3E-03	t
Cancer_Neoplastic_Met	6.5E-08	1.7E-06	t	Et743_Sarcoma_Dn	1.8E-04	7.9E-03	t
Canonical Pathways	p-val	q-val	Notes	Canonical Pathways	p-val	q-val	Notes
Propanoate_Metabolism	3.7E-02	1	bt	Proliferation_Genes	1.3E-02	1	c
Tryptophan_Metabolis	5.6E-02	1	t	Peptide_Gpcrs	2.2E-02	1	bt
Gpcrs_Class_A_Rhod	6.8E-02	1	bt	Ctla4pathway	3.2E-02	1	
Gpcrdb_Class_A_Rhod	8.1E-02	1	bt	Il17pathway	3.5E-02	1	bt
Beta_Alanine_Metaboli	8.7E-02	1		Cell_Proliferation	3.5E-02	1	c
Statin_Pathway_Pharmg	9.2E-02	1		Tcytotoxicpathway	3.8E-02	1	
Tyrosine_Metabolism	1.0E-01	1	bt	Tcrapathway	5.2E-02	1	
Alanine_And_Aspartate	1.0E-01	1		Intrinsicpathway	5.5E-02	1	bt
Nitrogen_Metabolism	1.1E-01	1	t	Breast_Cancer_Estrogen	5.5E-02	1	bt
Lairpathway	1.1E-01	1	Notes	Extrinsicpathway	6.1E-02	1	bt

Supplementary Table 5. GAGE applied to the two lung cancer datasets of large sample sizes following a grp-on-grp comparison scheme. Top 10 most significantly enriched experimental sets and canonical pathways in poor clinic outcomes vs good outcomes were inferred by GAGE from two published lung adenocarcinoma data sets used in GSEA paper [1]. Both positively and negatively regulated gene sets were collected and ranking by the p-value, and by absolute value of t statistics (not shown) for ties. Experiment and results description here are the same as Table 1, except that poor clinic outcomes are compare to good outcomes as a whole group using grp-on-grp comparison scheme rather than 1-on-grp. Experiment and results description here is the same as Table 1.

GAGE

Experiment Sets	p-val	q-val	Notes	Canonical Pathways	p-val	q-val	Notes
Rome_Insulin_2f_Up	4.4E-91	3.6E-88	d	Pgc	7.9E-64	2.7E-61	d
Mootha_Voxphos	7.6E-60	3.7E-57	d	Human_Mitodb_6_2002	6.4E-57	1.1E-54	d
Rcc_Nl_Up	1.2E-44	4.7E-42		Electron_Transport_Cha	1.3E-55	1.4E-53	d
Uvb_Nhek3_All	4.4E-43	1.2E-40		Mitochondria	2.2E-49	1.9E-47	d
Ventricles_Up	1.7E-41	3.4E-39		Ribosomal_Proteins	4.6E-35	3.1E-33	
Peng_Glutamine_Dn	8.2E-35	1.3E-32	m	Oxidative_Phosphorylati	7.3E-26	4.1E-24	d
Peng_Rapamycin_Dn	2.4E-29	3.3E-27	m	Striated_Muscle_Contra	7.2E-22	3.5E-20	m
Heartfailure_Atria_Dn	9.3E-24	1.1E-21		Proteasomepathway	2.7E-21	1.2E-19	
Peng_Leucine_Dn	1.3E-22	1.3E-20	m	Proteasome_Degradation	7.8E-20	2.9E-18	
Diab_Neph_Dn	1.7E-22	1.5E-20	d	Circadian_Exercise	6.0E-18	2.0E-16	m

PAGE

Experiment Sets	p-val	q-val	Notes	Canonical Pathways	p-val	q-val	Notes
Mootha_Voxphos	0	0	d	Ribosomal_Proteins	0	0	
Passerini_Complement	0	0		Electron_Transport_Cha	0	0	d
Igf_Vs_Pdgf_Up	0	0		Oxidative_Phosphorylati	0	0	d
Idx_Tsa_Up_Cluster6	0	0		Striated_Muscle_Contra	0	0	m
Hcc_Survival_Good_Vs	0	0		Human_Mitodb_6_2002	0	0	d
Ventricles_Up	0	0		Mitochondria	0	0	d
Aged_Mouse_Cortex_D	0	0		Cell2cellpathway	0	0	
Lvad_Heartfailure_Up	0	0		Ucalpainpathway	0	0	
Uvb_Nhek2_Up	0	0		Ubiquinone_Biosynthesi	0	0	
Goldrath_Hp	0	0		Pgc	0	0	d

GSEA

Experiment Sets	p-val	q-val	Notes	Canonical Pathways	p-val	q-val	Notes
Kannan_P53_Dn	<1E-03	1.2E-01		Tgfbpathway	8.4E-03	2.4E-01	m
Osawa_Tnfa_Hepatocyt	2.0E-03	2.8E-01	d	Caspasepathway	1.2E-02	4.4E-01	
Lizuka_L0_Gr_L1	1.3E-02	3.4E-01		Mitochondriapathway	1.7E-02	5.6E-01	m
P53genes_All	8.2E-03	3.8E-01		Ck1pathway	2.6E-02	6.5E-01	
Tsadac_Hypermeth_Ov	2.0E-03	5.4E-01		Tyrosine_Metabolism	4.2E-02	7.0E-01	m
Hpv31_Up	4.1E-03	5.5E-01		Sa_Programmed_Cell_D	6.1E-02	7.0E-01	
Hdaci_Colon_Tsa12hrs	1.2E-02	5.9E-01		Sturla_Sonic_Hedgehog	4.1E-02	7.2E-01	
Graeber_Beta2_Integrin	3.6E-02	7.6E-01		Agpcrpathway	1.9E-02	7.2E-01	
Insulin_Adip_Sens_Dn	1.3E-02	8.2E-01	d	St_T_Cell_Signal_Trans	7.0E-02	7.2E-01	
Vernell_Prb_Clstr1	2.5E-02	8.3E-01		Gata3pathway	4.2E-02	7.3E-01	

Supplementary Table 6. GAGE, PAGE or GSEA applied to the type 2 diabetes dataset of large sample size. Top 10 most significantly enriched experimental sets and canonical pathways in type 2 diabetes patients vs healthy controls were inferred by GAGE, PAGE or GSEA from the published data set generated by Mootha et al [2].Both positively and negatively regulated gene sets were collected and sorted by the p-value and by absolute value of t or z statistics (not shown) for ties for GAGE and PAGE, or sorted by the FDR value and by nominal p-value for ties for GSEA. Notes show the connections of the gene sets to type 2 diabetes, d stands for type 2 diabetes, m for metabolism related, blank represent no evident connection. These annotations came from the original studies for experimental sets, and from relevant literature for the canonical pathway. (0 suggests a positive value < 1E-324.)

Gene Sets & Methods		Top 10 P	Diabetes	Metab.	Sign. Calls
Experiment Sets	GAGE	1.7E-22	3	3	168 (184)
	PAGE	0 (<1.0E-324)	1	0	920 (954)
	GSEA	3.6E-2	2	0	1 (0)
Canonical Pathways	GAGE	6.0E-18	5	2	39 (44)
	PAGE	0 (<1.0E-324)	5	1	330 (344)
	GSEA	7.0E-2	0	3	0 (0)

Supplementary Table 7. Comparison between GAGE, PAGE and GSEA results from the type 2 diabetes dataset. The most significantly enriched experimental sets and canonical pathways in type 2 diabetes patients vs healthy controls were inferred by GAGE, PAGE and GSEA from published data set generated by Mootha et al [2]. Data columns are top 10 p-values, number of top 10 gene sets related to type 2 diabetes and metabolism, and numbers of significant gene sets with p-value ≤ 0.001 (or FDR q-value ≤ 0. 01).

Experiment Sets	z-statistic	p-value	q-value	P.exp1	P.exp2
Rett_Dn	37.2	2.6E-291	2.2E-288	1.1E-170	1.3E-233
Gh_Hypophysectomy_Rat_Up	30.7	2.2E-202	9.3E-200	6.1E-61	6.5E-281
Uvc_High_D2_Dn	29.1	3.2E-182	9.0E-180	4.1E-79	5.3E-195
Gh_Igf_Chondrocytes_Up	29.1	1.1E-181	2.3E-179	1.3E-77	4.7E-197
Passerini_Growth	-28.3	7.2E-173	4.4E-170	6.8E-96	1.4E-146
Ifna_Hcmv_6hrs_Up	-26.6	3.1E-153	9.6E-151	6.9E-86	1.8E-128
Lvad_Heartfailure_Dn	-25.8	6.8E-144	1.4E-141	3.1E-88	6.9E-109
Uvc_Low_C1_Dn	25.1	2.0E-136	3.4E-134	5.1E-89	1.5E-95
Baf57_Bt549_Dn	-25.0	5.3E-136	8.2E-134	2.5E-66	2.3E-131
Der_Ifnb_Up	-24.6	1.3E-131	1.6E-129	2.3E-64	8.1E-127
Canonical Pathways	z-statistic	p-value	q-value	P.exp1	P.exp2
Apoptosis	-14.9	3.6E-50	9.2E-48	9.4E-32	3.2E-37
Tgf_Beta_Signaling_Pathway	13.6	4.1E-42	1.3E-39	1.2E-26	1.8E-31
Valine_Leucine_And_Isoleucine_Degradation	-13.0	1.5E-38	2.0E-36	3.6E-32	2.4E-19
Striated_Muscle_Contraction	12.7	7.2E-37	1.1E-34	2.5E-20	1.8E-32
Tob1pathway	-12.5	5.6E-36	4.7E-34	2.0E-25	1.9E-23
Gpcrdb_Other	12.4	1.0E-35	1.0E-33	4.7E-12	3.7E-48
Badpathway	11.8	1.7E-32	1.3E-30	7.4E-20	1.2E-25
Mitochondria	-11.6	2.9E-31	1.8E-29	9.9E-09	4.6E-48
Eicosanoid_Synthesis	11.5	5.7E-31	3.4E-29	7.7E-13	5.5E-36
Apoptosis_Genmapp	-10.9	8.8E-28	4.5E-26	2.2E-12	1.5E-30

Supplementary Table 8. PAGE applied to the BMP6-MSC dataset of small sample size. Top 10 most significantly differentially expressed experimental sets and canonical pathways in human MSC following 8 hour BMP6 treatment were inferred by PAGE. PAGE by default apples to whole data set with replicate samples and gave the global p-value. Upon small modification to enable one-on-one comparison, PAGE was applied to each of the two BMP6 experiments individually the same way as GAGE in Table 3. Note that PAGE originally included no p-value adjustment for the multiple testing issue, we added FDR procedure to PAGE here for comparison purpose.

Experiment Sets	NES	Nom. P	FDR
Ifna_Hcmv_6hrs_Up	-2.37	<1.00E-03	0
Lvad_Heartfailure_Dn	-2.22	<1.00E-03	0
Der_Ifnb_Up	-2.17	<1.00E-03	0
Ifn_Beta_Up	-2.16	<1.00E-03	0
Ifna_Uv-Cmv_Common_Hcmv_6hrs_Up	-2.12	<1.00E-03	0.002
Hif1_Targets	-2.03	<1.00E-03	0.007
Zhan_Mmpc_Simal	-2.01	<1.00E-03	0.007
Dac_Bladder_Up	-2.02	<1.00E-03	0.007
Ifn_Any_Up	-1.98	<1.00E-03	0.011
Dac_Ifn_Bladder_Up	-1.97	<1.00E-03	0.013
Canonical Pathways	NES	Nom. P	FDR
Valine_Leucine_And_Isoleucine_Degradation	-1.80	2.03E-03	0.241
Deathpathway	-1.80	<1.00E-03	0.357
Apoptosis	-1.83	<1.00E-03	0.452
Propanoate_Metabolism	-1.63	1.55E-02	0.651
Trna_Synthetases	-1.60	3.49E-02	0.681
Apoptosis_Genmapp	-1.64	5.73E-03	0.717
Striated_Muscle_Contraction	1.59	2.41E-02	0.736
Ranklpathway	-1.58	3.70E-02	0.743
Gpcrdb_Other	1.55	2.76E-02	0.745
Pitx2pathway	1.53	6.81E-02	0.752

Supplementary Table 9. GSEA-g (permutation of gene labels) applied to the BMP6-MSC dataset of small sample size. Top 10 most significantly differentially expressed experimental sets and canonical pathways in human MSC following 8 hour BMP6 treatment were inferred by GSEA-g. Both positively and negatively regulated gene sets were collected and ranking by the FDR q-value, and by the nominal p-values and then absolute NES for ties.

Gene Sets & Methods		GAGE	PAGE	GSEA
Experiment Sets	GAGE	NA	3	6
	PAGE	3	NA	3
	GSEA-g	6	3	NA
Canonical Pathways	GAGE	NA	2	1
	PAGE	2	NA	5
	GSEA-g	1	5	NA

Supplementary Table 10. Overlaps between GAGE, PAGE and GSEA-g results from the BMP6-MSC dataset. The top 10 most significantly differentially expressed experimental sets experimental sets and canonical pathways in human MSC following 8 hour BMP6 treatment were inferred by GAGE, PAGE and GSEA-g.

Experiment Sets	t-statistic	p-value	q-value	P.exp1	P.exp2
Cmv_Hcmv_Timecourse_All_Dn	5.81	9.3E-16	5.4E-13	1.7E-09	1.4E-08
Uvc_High_All_Dn	4.21	4.3E-09	9.1E-07	3.0E-05	6.1E-06
Baf57_Bt549_Dn	4.23	4.8E-09	9.4E-07	2.0E-06	1.0E-04
Baf57_Bt549_Up	4.09	1.5E-08	2.1E-06	8.5E-05	7.7E-06
Cmv_Hcmv_6hrs_Dn	3.98	1.5E-07	1.7E-05	5.2E-05	1.4E-04
Takeda_Nup8_Hoxa9_3d_Up	3.71	2.8E-07	2.6E-05	5.2E-04	2.8E-05
Cmv_Hcmv_Timecourse_All_Up	3.61	4.8E-07	3.6E-05	2.6E-04	1.0E-04
Li_Fetal_Vs_Wt_Kidney_Up	3.61	5.7E-07	3.8E-05	1.7E-04	1.8E-04
Cmv-Uv_Hcmv_6hrs_Up	3.63	6.0E-07	3.9E-05	3.1E-04	1.1E-04
Boquest_Cd31plus_Vs_Cd31minus_Dn	3.48	1.2E-06	7.2E-05	6.4E-04	1.1E-04
**Canonical Pathways**	**t-statistic**	**p-value**	**q-value**	**P.exp1**	**P.exp2**
Valine_Leucine_And_Isoleucine_Degradation	-2.32	1.3E-03	1	4.3E-03	3.0E-02
Mitochondria	-2.15	1.3E-03	1	9.6E-02	1.4E-03
Apoptosis	-2.07	3.6E-03	1	1.8E-02	2.3E-02
Propanoate_Metabolism	-1.69	1.3E-02	1	1.2E-02	1.5E-01
Gpcrdb_Other	1.67	1.4E-02	1	1.3E-01	1.4E-02
Human_Mitodb_6_2002	-1.40	2.3E-02	1	3.1E-01	1.1E-02
Apoptosis_Genmapp	-1.53	2.6E-02	1	1.0E-01	3.9E-02
Limonene_And_Pinene_Degradation	-1.56	2.7E-02	1	3.3E-02	1.3E-01
Beta_Alanine_Metabolism	-1.46	3.0E-02	1	2.7E-02	1.8E-01
Raspathway	-1.46	3.6E-02	1	7.2E-02	8.1E-02

Supplementary Table 11. GAGE with the opposite assumption on canonical pathways vs experimental sets applied to the BMP6-MSC dataset. Top 10 most significantly differentially expressed experimental sets and canonical pathways in human MSC following 8 hour BMP6 treatment were inferred by GAGE with the exact opposite assumption that all genes in a canonical pathways are regulated towards the same direction, either up or down, whereas genes in an experimental set can be regulated towards both directions at the same time. This analysis is the same as that for Table 3 otherwise.

Experiment Sets	z-statistic	p-value	q-value	P.exp1	P.exp2
Rett_Dn	30.6	0	0	1.1E-170	1.3E-233
Gh_Hypophysectomy_Rat_Up	26.5	0	0	6.1E-61	6.5E-281
Gh_Igf_Chondrocytes_Up	24.5	3.8E-271	9.7E-269	1.3E-77	4.7E-197
Uvc_High_D2_Dn	24.5	1.4E-270	2.6E-268	4.1E-79	5.3E-195
Passerini_Growth	-23.4	5.3E-239	2.8E-236	6.8E-96	1.4E-146
Ifna_Hcmv_6hrs_Up	-22.0	6.0E-211	1.6E-208	6.9E-86	1.8E-128
Baf57_Bt549_Dn	-20.9	2.6E-194	4.5E-192	2.5E-66	2.3E-131
Lvad_Heartfailure_Dn	-21.1	9.8E-194	1.3E-191	3.1E-88	6.9E-109
Bcrabl_Hl60_Cdna_Up	20.0	4.2E-189	6.4E-187	6.6E-43	1.4E-149
Der_Ifnb_Up	-20.5	8.1E-188	8.4E-186	2.3E-64	8.1E-127
Canonical Pathways	z-statistic	p-value	q-value	P.exp1	P.exp2
Tgf_Beta_Signaling_Pathway	48.9	0	0	0	0
Alkpathway	39.5	0	0	4.9E-291	0
Wnt_Signaling	23.7	4.1E-244	3.6E-242	7.6E-153	9.6E-95
Erythpathway	19.6	9.9E-167	6.4E-165	3.4E-83	7.5E-87
Proliferation_Genes	19.6	1.8E-166	9.5E-165	1.7E-91	2.8E-78
Eicosanoid_Synthesis	17.6	3.1E-145	1.4E-143	2.5E-36	3.7E-112
Smooth_Muscle_Contraction	17.9	2.5E-139	9.2E-138	4.2E-75	1.8E-67
Cell_Proliferation	17.7	6.5E-139	2.2E-137	4.1E-55	5.0E-87
Hematopoesis_Related_Transcription_Factors	17.5	4.4E-135	1.3E-133	3.4E-54	4.0E-84
Breast_Cancer_Estrogen_Signaling	16.3	3.4E-124	9.1E-123	1.7E-32	6.8E-95

Supplementary Table 12. GAGE-z (with one-sample z test option) applied to the BMP6-MSC dataset. Top 10 most significantly differentially expressed experimental sets and canonical pathways in human MSC following 8 hour BMP6 treatment were inferred by GAGE-z which uses one-sample z-test instead of the two-sample t-test for the gene set significance as in GAGE. GAGE-z is a GAGE style version of PAGE, which ensembles GAGE in gene set regulation direction assumption and one-on-one comparison. (0 suggests a positive value < 1E-324.)

Experiment Sets	t-statistic	p-value	q-value	P.exp1	P.exp2
Ifna_Hcmv_6hrs_Up	-3.88	1.7E-07	1.4E-04	2.3E-04	3.8E-05
Der_Ifnb_Up	-3.33	2.9E-06	1.2E-03	6.1E-03	2.8E-05
Grandvaux_Ifn_Not_Irf3_Up	-3.69	1.1E-05	2.6E-03	9.7E-03	7.9E-05
Baf57_Bt549_Dn	-3.02	1.3E-05	2.7E-03	1.7E-02	5.4E-05
Dac_Bladder_Up	-2.95	5.2E-05	6.5E-03	1.6E-04	2.4E-02
Ifn_Any_Up	-2.86	5.4E-05	6.6E-03	1.5E-02	2.7E-04
Ifn_Beta_Up	-2.86	6.1E-05	7.0E-03	1.4E-02	3.2E-04
Cmv_Hcmv_Timecourse_All_Up	-2.57	7.3E-05	7.5E-03	1.0E-01	5.3E-05
Sana_Ifng_Endothelial_Up	-2.77	1.2E-04	1.1E-02	7.2E-03	1.4E-03
Li_Fetal_Vs_Wt_Kidney_Up	-2.61	2.5E-04	2.0E-02	9.1E-03	2.3E-03
Canonical Pathways	t-statistic	p-value	q-value	P.exp1	P.exp2
Tgf_Beta_Signaling_Pathway	3.27	7.2E-06	2.8E-03	1.9E-04	2.5E-03
Alkpathway	2.74	1.8E-04	3.5E-02	1.7E-03	8.9E-03
Ganglioside_Biosynthesis	2.30	2.2E-03	2.7E-01	1.3E-02	1.8E-02
Erythpathway	2.17	3.4E-03	3.4E-01	3.6E-02	1.1E-02
Hematopoesis_Related_Transcription_Factors	2.04	3.6E-03	3.5E-01	7.8E-03	5.1E-02
Cd40pathway	2.09	3.9E-03	3.7E-01	5.0E-03	9.0E-02
Tnfr2pathway	1.98	5.8E-03	4.7E-01	9.2E-03	7.7E-02
Hypertrophy_Model	1.78	1.1E-02	6.2E-01	1.3E-02	1.1E-01
Apoptosis	1.72	1.2E-02	6.5E-01	9.2E-02	1.8E-02
St_Tumor_Necrosis_Factor_Pathway	1.71	1.3E-02	6.6E-01	1.3E-01	1.4E-02

Supplementary Table 13. GAGE-r (with rank-test option) applied to the BMP6-MSC dataset. Top 10 most significantly differentially expressed experimental sets and canonical pathways in human MSC following 8 hour BMP6 treatment were inferred by GAGE-r, which uses a rank-based two-sample test instead of the default parametric two-sample t-test for the gene set significance as in GAGE. This analysis is the same as that for Table 3 otherwise.

Experiment Sets	t-statistic	p-value	q-value
Ifna_Hcmv_6hrs_Up	-4.17	3.3E-05	1.8E-02
Der_Ifnb_Up	-3.75	1.2E-04	3.3E-02
Baf57_Bt549_Dn	-3.31	5.0E-04	9.0E-02
Ifn_Beta_Up	-3.16	1.0E-03	1.2E-01
Sana_Ifng_Endothelial_Up	-3.10	1.2E-03	1.3E-01
Ifn_Any_Up	-2.96	1.8E-03	1.6E-01
Grandvaux_Ifn_Not_Irf3_Up	-3.07	2.5E-03	1.9E-01
Dac_Bladder_Up	-2.88	2.9E-03	2.0E-01
Ifna_Uv-Cmv_Common_Hcmv_6hrs_Up	-2.78	3.8E-03	2.2E-01
Bennett_Sle_Up	-2.69	4.8E-03	2.5E-01
**Canonical Pathways**	**t-statistic**	**p-value**	**q-value**
Tgf_Beta_Signaling_Pathway	3.24	9.5E-04	1
Wnt_Signaling	2.83	2.8E-03	1
Proliferation_Genes	2.65	4.2E-03	1
Alkpathway	2.52	7.9E-03	1
Cell_Proliferation	2.30	1.1E-02	1
Hematopoesis_Related_Transcription_Factors	2.20	1.5E-02	1
Smooth_Muscle_Contraction	2.11	1.8E-02	1
Erythpathway	2.23	1.9E-02	1
Ganglioside_Biosynthesis	2.15	2.1E-02	1
Apoptosis	1.93	2.8E-02	1

Supplementary Table 14. GAGE applied to the BMP6-MSC dataset following a grp-on-grp comparison scheme. Top 10 most significantly differentially expressed experimental sets and canonical pathways in human MSC following 8 hour BMP6 treatment were inferred by GAGE following grp-on-grp comparison scheme rather than the 1-on-1 paired comparison scheme. This analysis is the same as that for Table 3 otherwise.

Boston

Gene Sets & Methods		1-on-1	1-on-grp	grp-on-grp
Experiment Sets	1-on-1	NA	9	7
	1-on-grp	9	NA	8
	grp-on-grp	7	8	NA
Canonical Pathways	1-on-1	NA	8	4
	1-on-grp	8	NA	3
	grp-on-grp	4	3	NA

Michigan

Gene Sets & Methods		1-on-1	1-on-grp	grp-on-grp
Experiment Sets	1-on-1	NA	7	5
	1-on-grp	7	NA	7
	grp-on-grp	5	7	NA
Canonical Pathways	1-on-1	NA	9	5
	1-on-grp	9	NA	4
	grp-on-grp	5	4	NA

Supplementary Table 15. Overlaps between results inferred using three comparison schemes of GAGE, 1-on-1, 1-on-grp and grp-on-grp, from the two lung adenocarcinoma datasets. Top 10 most significantly enriched experimental sets and canonical pathways in poor clinic outcomes vs good outcomes were inferred by GAGE with different comparison schemes, 1-on-1, 1-on-grp and grp-on-grp, from the two published lung adenocarcinoma datasets used in the GSEA paper [1].

Supplementary Figures

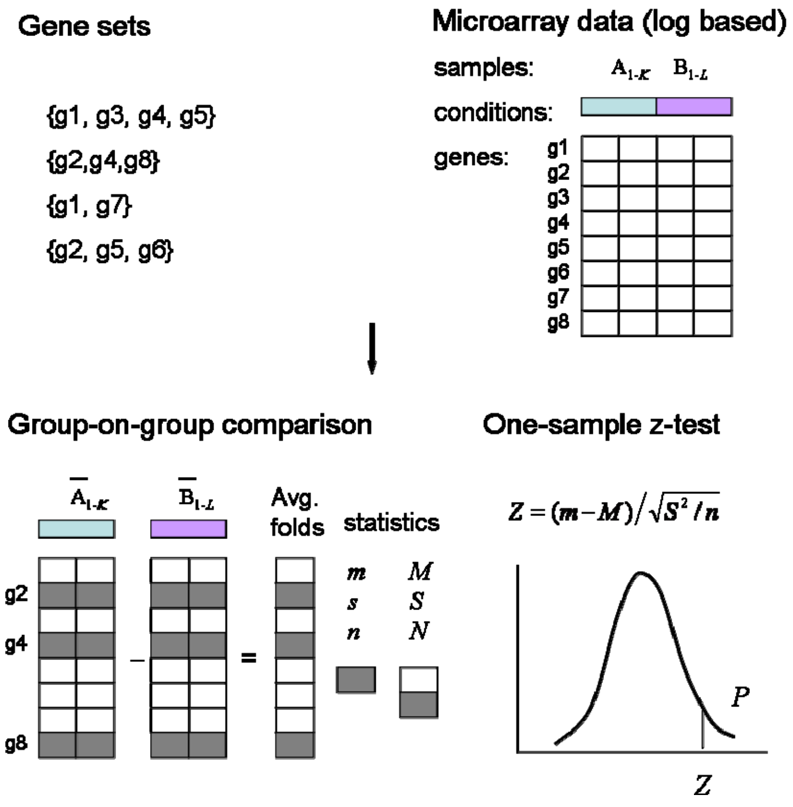

Supplementary Figure 1. A schematic overview of PAGE procedure [3]. Unlike GSEA [1] but similar to GAGE (Figure 1), PAGE is a parametric method. While GSEA estimate significance of gene set differential expression using permutation of sample labels, PAGE does one-sample z-tests since the log based mean fold change of a gene set closely follows a normal distribution [3].Note that expression levels are log transformed. Variables *m*, *s* and *n* are the mean fold change, standard deviation and number of genes in a gene set, *M*, *S* and *N* are those for the whole set.

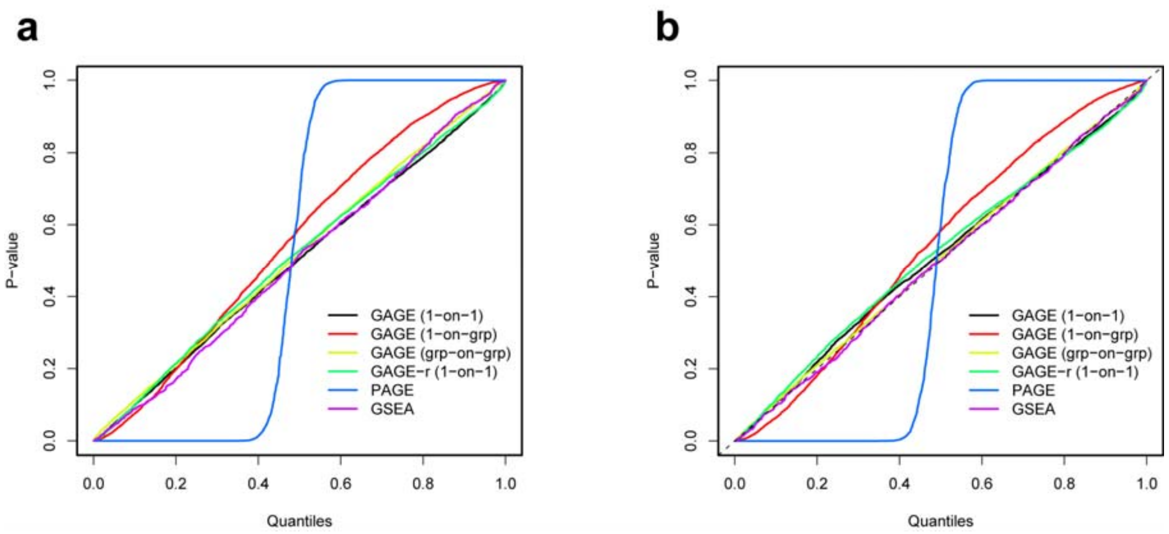

Supplementary Figure 2. The p-value distributions for GAGE, GSEA and PAGE under the null condition in the simulation study. (a) gene set size =10 and (b) gene set size =50. For GAGE, all three comparison schemes, i.e. 1-on-1, 1-on-grp and grp-on-grp, and two different tests, i.e. parametric two-sample t-test (default) and ranked based t-test (GAGE-r) were examined. To control the null condition, sample labels were randomly permuted and gene sets of 10 genes or 50 genes were randomly sampled (i.e. β= α =1). To make sure strictly null condition, the gene set was compared to the whole set instead of virtual random set of the same size (details in Significance test subsection of Methods) in GAGE since the testing gene sets are sampled from the whole set. Each experiment was carried out 5000 times in total.

Supplementary Notes

Supplementary Note 1

T-profiler employs two-sample t-test, but it compares a gene set to the complementary set of all other genes, and assumes equal variance between the two set, which made it similar to a one-sample z-test in PAGE. In the formulas of the T-profiler [4], given $N_{G'}$ is much greater than N_G, the pooled standard error s approximately equal $s_{G'}$, and t statistics essentially equals z statistics.

Supplementary Note 2

GAGE pinpointed multiple experimental sets and canonical pathways which are directly involved in type 2 diabetes or closely related metabolism processes. PAGE and GSEA inferred fewer relevant top gene sets. Interestingly, the two gold standard oxidative phosphorylation gene sets, Mootha_Voxphos and Oxidative_Phosphorylation, were selected as top 2 experimental set and top 6 canonical pathway by GAGE. But they ranked out of top 10 by PAGE and GAGE despite they were listed as the most significant gene sets in the original GSEA [2] and PAGE studies [3]. This discrepancy can be attributed to two facts: first, we used the more comprehensive and updated curated gene set collection c2 [1] which includes more relevant gene sets ranking on the top; second, we used an updated version of GSEA [1], which included a normalization step to eliminate the effect of different gene set sizes hence is different from the first version of GSEA [2].

Supplementary Note 3

Note that we used GAGE program to remove PAGE redundant gene sets since PAGE does not offer such function. This remover program has been optimized for GAGE, where there were no or very few false positive calls. When applied to PAGE results, the large number of false positive calls may result in excessive redundancy removal, hence the non-redundant list could be shorter than it should be. Nonetheless, the non-redundant list is a good reference for the comparison between GAGE and PAGE.

Supplementary Note 4

GAGE more stresses the overall expression changes of the whole set, whereas PAGE is more sensitive to big changes of individual genes. GAGE can be considered more competitive (Q1) and PAGE more like self-contained (Q2) according to the classification described by Geoman [5] and Nam [6], although both are assigned to the big competitive (Q1) category.

Supplementary Note 5

First, for two groups each with n samples, we can do n independent-tests on sample pairs, yet only one test on the group averages. Obviously, the former is more powerful than the latter. Second, for gene set based analyses, what really matters is the change for the whole gene set not that for individual genes. Hence big fluctuation for single gene expression level is considered common for the same experiment condition (or within group variance) as long as the whole set net effect is zero. Taking average of such fluctuated gene expression levels within the sample group as the representative expression level would be misleading when the net effect of the gene set is non-additive as seen in many canonical pathways (where we take set mean of the absolute fold changes). Take a simplified example, we have a gene set of two genes, the expression level for the control condition is (2, 2). This set is perturbed for the two samples under experiment condition and becomes (4, 0) and (0, 4), both are able to achieve certain effect because the two genes are functionally related (like A OR B but not A AND B). But the average over the experimental condition is (2, 2), no different than the control at all.

References

1. Subramanian A, Tamayo P, Mootha VK, Mukherjee S, Ebert BL, Gillette MA, Paulovich A, Pomeroy SL, Golub TR, Lander ES, et al: **Gene set enrichment analysis: a knowledge-based approach for interpreting genome-wide expression profiles**. *Proc Natl Acad Sci U S A* 2005, **102**:15545-50.
2. Mootha VK, Lindgren CM, Eriksson KF, Subramanian A, Sihag S, Lehar J, Puigserver P, Carlsson E, Ridderstrale M, Laurila E, et al: **PGC-1alpha-responsive genes involved in oxidative phosphorylation are coordinately downregulated in human diabetes**. *Nat Genet* 2003, **34**:267-73.
3. Kim SY, Volsky DJ: **PAGE: parametric analysis of gene set enrichment**. *BMC Bioinformatics* 2005, **6**:144.
4. Boorsma A, Foat BC, Vis D, Klis F, Bussemaker HJ: **T-profiler: scoring the activity of predefined groups of genes using gene expression data**. *Nucleic Acids Res* 2005, **33**:W592-5.
5. Goeman JJ, Buhlmann P: **Analyzing gene expression data in terms of gene sets: methodological issues**. *Bioinformatics* 2007, **23**:980-7.
6. Nam D, Kim SY: **Gene-set approach for expression pattern analysis**. *Brief Bioinform* 2008.
